# Genome-wide enrichment analysis between endometriosis and obesity-related traits reveals novel susceptibility loci

**DOI:** 10.1093/hmg/ddu516

**Published:** 2014-10-08

**Authors:** Nilufer Rahmioglu, Stuart Macgregor, Alexander W. Drong, Åsa K. Hedman, Holly R. Harris, Joshua C. Randall, Inga Prokopenko, The International Endogene Consortium (IEC), The GIANT Consortium, Dale R. Nyholt, Andrew P. Morris, Grant W. Montgomery, Stacey A. Missmer, Cecilia M. Lindgren, Krina T. Zondervan

**Affiliations:** 1 Wellcome Trust Center for Human Genetics, Universityof Oxford, Oxford OX3 7BN, UK,; 2 Statistical Genetics,; 3 Neurogenetics,; 4 Molecular Epidemiology, QIMR Berghofer Medical Research Institute, Brisbane, QLD 4029, Australia,; 5 Department of Medical Sciences, Molecular Epidemiology and Science for Life Laboratory, Uppsala University, Uppsala, Sweden,; 6 Department of Obstetrics, Gynecology and Reproductive Biology, Brigham and Women's Hospital and Harvard Medical School, 75 Francis Street, Boston, MA 02115, USA,; 7 Unit of Nutritional Epidemiology, Institute for Environmental Medicine, Karolinska Institutet, PO Box 210, SE-171 77 Stockholm, Sweden,; 8 Wellcome Trust Sanger Institute, Hinxton, Cambridge CB10 1SA, UK,; 9 Department of Genomics of Common Disease, Imperial College London, London W12 0NN, UK,; 10 Oxford Centre for Diabetes, Endocrinology and Metabolism, University of Oxford, Oxford OX3 7LJ, UK,; 11 Department of Biostatistics, University of Liverpool, Duncan Building, Daulby Street, Liverpool L69 3GA, UK,; 12 Broad Institute of the Massachusetts Institute of Technology and Harvard University, Cambridge02142 MA, USA and; 13 Nuffield Department of Obstetrics and Gynaecology & Endometriosis CaRe Centre, University of Oxford, John Radcliffe Hospital, Oxford OX3 9DU, UK

## Abstract

Endometriosis is a chronic inflammatory condition in women that results in pelvic pain and subfertility, and has been associated with decreased body mass index (BMI). Genetic variants contributing to the heritable component have started to emerge from genome-wide association studies (GWAS), although the majority remain unknown. Unexpectedly, we observed an intergenic locus on 7p15.2 that was genome-wide significantly associated with both endometriosis and fat distribution (waist-to-hip ratio adjusted for BMI; WHRadjBMI) in an independent meta-GWAS of European ancestry individuals. This led us to investigate the potential overlap in genetic variants underlying the aetiology of endometriosis, WHRadjBMI and BMI using GWAS data. Our analyses demonstrated significant enrichment of common variants between fat distribution and endometriosis (*P* = 3.7 × 10^−3^), which was stronger when we restricted the investigation to more severe (Stage B) cases (*P* = 4.5 × 10^−4^). However, no genetic enrichment was observed between endometriosis and BMI (*P* = 0.79). In addition to 7p15.2, we identify four more variants with statistically significant evidence of involvement in both endometriosis and WHRadjBMI (in/near *KIFAP3*, *CAB39L*, *WNT4*, *GRB14*); two of these, *KIFAP3* and *CAB39L*, are novel associations for both traits. *KIFAP3*, *WNT4* and 7p15.2 are associated with the *WNT* signalling pathway; formal pathway analysis confirmed a statistically significant (*P* = 6.41 × 10^−4^) overrepresentation of shared associations in developmental processes/*WNT* signalling between the two traits. Our results demonstrate an example of potential biological pleiotropy that was hitherto unknown, and represent an opportunity for functional follow-up of loci and further cross-phenotype comparisons to assess how fat distribution and endometriosis pathogenesis research fields can inform each other.

## INTRODUCTION

Endometriosis is a common condition in premenopausal women characterized by chronic pelvic inflammation causing pain and subfertility (1), and has an estimated heritability of 51% (2). The International Endogene Consortium (IEC) performed the largest endometriosis GWAS to date in 3194 surgically confirmed cases (including 1364 moderate–severe—Stage B—cases) and 7060 controls of European ancestry, with replication in a further 2392 cases and 2271 controls (3). One genome-wide significant locus was observed in an intergenic region on chromosome 7p15.2 (rs12700667), primarily associated with Stage B disease (*P* = 1.5 × 10^−9^, OR = 1.38, 95% CI 1.24–1.53). A second locus near *WNT4* (rs7521902) was found after meta-analysis with published results from a Japanese GWAS of 1423 cases and 1318 controls (4); a genome-wide meta-analysis confirmed the two loci and found a further five (5).

Rs12700667 on 7p15.2 also marked 1 of 16 reported genome-wide significant loci associated with waist-to-hip ratio adjusted for BMI (WHRadjBMI) in an independent GWAS meta-analysis by the GIANT Consortium involving 77 167 individuals of European ancestry with replication in a further 113 636 individuals (rs1055144: discovery *P* = 1.5 × 10^−8^; meta-analysis *P* = 1.0 × 10^−24^; *r*^2^ = 0.5 with rs12700667 in 1000G pilot CEU data) (6,7). This was surprising, as prospective epidemiological studies have suggested consistently that reduced BMI—a measure of overall adiposity—is associated with increased risk of endometriosis, but there is relatively limited evidence for an association with WHRadjBMI—a measure of fat distribution (8,9). We conducted a logistic regression analysis in the IEC dataset of rs1055144 on endometriosis disease status, conditioning on rs12700667, which demonstrated that the SNPs reflected the same association signal (unpublished data; conditional *P* = 0.65).

The epidemiological evidence of an association between endometriosis and BMI, together with the observed GWAS locus in common between endometriosis and WHRadjBMI, led us to conduct a systematic investigation of overlap in association signals between the IEC endometriosis GWAS and GIANT Consortium WHRadjBMI (*N* = 77 167) (6,7) and BMI (*N* = 123 865) (7,10) meta-GWAS datasets through genetic enrichment analyses.

## RESULTS

### Genetic enrichment analysis of endometriosis with overall adiposity and fat distribution

Using independent, imputed (1000 Genomes pilot reference panel) GWAS datasets of endometriosis (IEC; 3194 cases including 1364 Stage B cases, 7060 controls), BMI (GIANT; 123 865 individuals) and WHRadjBMI (GIANT: 77 167 individuals), we first considered loci genome-wide significantly associated with endometriosis, BMI or WHRadjBMI in each of the individual GWAS. The two genome-wide significant endometriosis loci (intergenic 7p15.2 and *WNT4*) had significantly lower *P*-values of association than expected by chance in the WHRadjBMI GWAS (Table 1: rs12700667, *P* = 4.4 × 10^−5^ and rs7521902, *P* = 1.3 × 10^−3^; binomial *P* = 1.0 × 10^−4^), while 2 of the 16 genome-wide significant WHRadjBMI loci (intergenic 7p15.2 and *GRB14*) had *P* < 0.01 in the endometriosis GWAS (binomial *P* = 0.011). No enrichment between genome-wide significantly associated loci was observed for endometriosis versus BMI (Supplementary Material, Table S1: rs12700667, *P* = 0.27 and rs7521902, *P* = 0.92).

**Table 1.** Association results of published IEC genome-wide significant endometriosis loci (3) in the GIANT WHRadjBMI GWAS, and of WHRadjBMI loci (6,7) in endometriosis GWAS (lookup results are shown in bold)

GWAS	SNP (proxy; *r*^2^)	Ch	Location (B36)	RAF (allele)	Status	Endometriosis all cases		Endometriosis Stage B only		Overall WHRadjBMI		Female-limited WHRadjBMI		Nearest gene
						*P*-value^c^	OR (95% CI)	*P*-value^c^	OR (95% CI)	*P*-value^d^	Effect (SE)	*P*-value^e^	Effect (SE)	
Endometriosis	rs12700667	7	25 868 164	0.74 (A)	G	5.1 × 10^−7^	1.21 (1.12–1.31)	3.3 × 10^−8^	1.36 (1.23–1.50)	**4.4 × 10^−5^**	−**0.023 (0.005)**	**3.3 × 10^−8^**	−**0.023 (0.005)**	Intergenic
Endometriosis	rs7521902	1	22 363 311	0.25 (A)	G	8.9 × 10^−5^	1.16 (1.08–1.25)	7.5 × 10^−5^	1.26 (1.14–1.39)	**1.3 × 10^−3^**	−**0.020 (0.006)**	**6.1 × 10^−3^**	−**0.023 (0.009)**	*WNT4*
WHRadjBMI	rs1055144^a^	7	25 837 634	0.19 (T)	G	**3.7 × 10^−5^**	**0.84 (0.77–0.91)**	3.1 × 10^−4^	0.78 (0.70–0.88)	1.5 × 10^−8^	0.034 (0.006)	2.3 × 10^−6^	0.039 (0.008)	Intergenic
WHRadjBMI	rs10195252	2	165 221 337	0.41 (C)	G	**9.8 × 10^−3^**	**0.92 (0.85–0.98)**	0.56	0.92 (0.84–1.00)	3.2 × 10^−10^	−0.031 (0.005)	6.3 × 10^−15^	−0.053 (0.007)	*GRB14*
Female WHRadjBMI	rs4684854	3	12 463 882	0.43 (C)	I (0.98)	**0.07**	**1.06 (0.99–1.14)**	0.14	1.07 (0.98–1.17)	1.0 × 10^−4^	0.019 (0.005)	2.3 × 10^−8^	0.039 (0.007)	*PPARG*
WHRadjBMI	rs718314	12	26 344 550	0.24 (G)	G	**0.11**	**1.06 (0.99–1.15)**	0.054	1.10 (0.99–1.22)	2.4 × 10^−8^	0.031 (0.005)	8.2 × 10^−10^	0.047 (0.008)	*ITPR2-SSPN*
WHRadjBMI	rs6861681	5	173 362 458	0.32 (A)	I (0.96)	**0.15**	**0.95 (0.86–1.04)**	0.11	0.93 (0.85–1.00)	1.4 × 10^−6^	0.026 (0.005)	2.1 × 10^−4^	0.027 (0.007)	*CPEB4*
WHRadjBMI	rs6795735	3	64 680 405	0.41 (T)	G	**0.21**	**1.04 (0.98–1.12)**	0.32	1.04 (0.96–1.14)	2.5 × 10^−7^	−0.025 (0.005)	7.8 × 10^−7^	−0.033 (0.007)	*ADAMTS9*
WHRadjBMI	rs2820446 (rs4846567, *r*^2^ = 1)^b^	1	21 974 881	0.71 (C)	I (0.99)	**0.31**	**1.04 (0.97–1.12)**	0.22	1.06 (0.97–1.17)	5.1 × 10^−12^	0.037 (0.005)	8.5 × 10^−18^	0.064 (0.007)	*LYPLAL1*
WHRadjBMI	rs498778 (rs6784615, *r*^2^ = 1)^b^	3	52 453 893	0.93 (T)	I (0.95)	**0.32**	**1.08 (0.93–1.24)**	0.25	1.06 (0.89–1.27)	4.6 × 10^−5^	0.055 (0.010)	1.1 × 10^−3^	0.063 (0.019)	*NISCH-STAB1*
WHRadjBMI	rs1294421	6	6 743 149	0.39 (T)	I (0.96)	**0.37**	**1.03 (0.94–1.10)**	0.28	1.03 (0.94–1.13)	6.3 × 10^−9^	−0.029 (0.005)	3.4 × 10^−8^	−0.038 (0.007)	*LY86*
WHRadjBMI	rs9491696	6	127 452 639	0.51 (C)	I (0.99)	**0.43**	**0.97 (0.91–1.03)**	0.64	0.98 (0.90–1.06)	2.1 × 10^−14^	−0.037 (0.005)	3.4 × 10^−8^	−0.038 (0.007)	*RSPO3*
WHRadjBMI	rs1443512	12	52 628 951	0.22 (A)	G	**0.62**	**1.02 (0.94–1.10)**	0.63	0.97 (0.88–1.08)	3.3 × 10^−8^	0.031 (0.005)	1.4 × 10^−9^	0.048 (0.008)	*HOXC13*
WHRadjBMI	rs984222	1	119 305 366	0.39 (C)	I (0.99)	**0.69**	**0.99 (0.93–1.05)**	0.31	0.95 (0.87–1.04)	3.8 × 10^−14^	−0.037 (0.005)	1.2 × 10^−7^	−0.036 (0.007)	*TBX15-WARS2*
WHRadjBMI	rs4823006	22	29 451 671	0.57 (A)	I (0.97)	**0.72**	**1.01 (0.95–1.08)**	0.82	1.01 (0.92–1.11)	4.7 × 10^−10^	0.030 (0.005)	6.9 × 10^−8^	0.037 (0.007)	*ZNRF3*
Female WHRadjBMI	rs10478424	5	118 816 619	0.79 (A)	I (0.97)	**0.80**	**1.01 (0.93–1.10)**	0.56	1.03 (0.93–1.15)	1.6 × 10^−4^	0.023 (0.006)	1.0 × 10^−5^	0.037 (0.009)	*HSD17B4*
WHRadjBMI	rs1011731	1	170 613 171	0.44 (G)	G	**0.81**	**0.99 (0.93–1.05)**	0.77	1.01 (0.93–1.11)	1.7 × 10^−10^	0.031 (0.005)	2.1 × 10^−5^	0.028 (0.007)	*DNM3-PIGC*
WHRadjBMI	rs6905288	6	43 866 851	0.56 (A)	I (0.80)	**0.66**	**0.98 (0.91–1.05)**	0.66	0.99 (0.90–1.08)	4.2 × 10^−10^	0.033 (0.005)	7.7 × 10^−13^	0.052 (0.007)	*VEGFA*

a Logistic regression analysis in the IEC GWAS shows that rs1055144 marks the same locus as rs12700667 (conditional *P* = 0.65; *r*^2^ = 0.8).

b SNP was not genotyped in the endometriosis GWAS dataset; result shown is of proxy SNP.

c Results are based on an updated GWAS performed using genotype data imputed up to 1000 Genomes pilot reference panel (B36, June 2010).

d Results are from the GIANT WHRadjBMI discovery GWAS dataset (*N* = 77 167); 3 of the 14 WHRadjBMI loci have *P* > 5.0 × 10^−8^, however, they reached genome-wide significance combined with replication analyses in up to a further 113 636 individuals (6).

e Results from the GIANT WHRadjBMI discovery female-limited GWAS dataset (*N* = 42 969); one of the two female-limited WHRadjBMI loci have *P* > 5.0 × 10^−8^, however, they reached genome-wide significance combined with replication analyses in up to a further 71 295 individuals (7).

**Figure 1.**
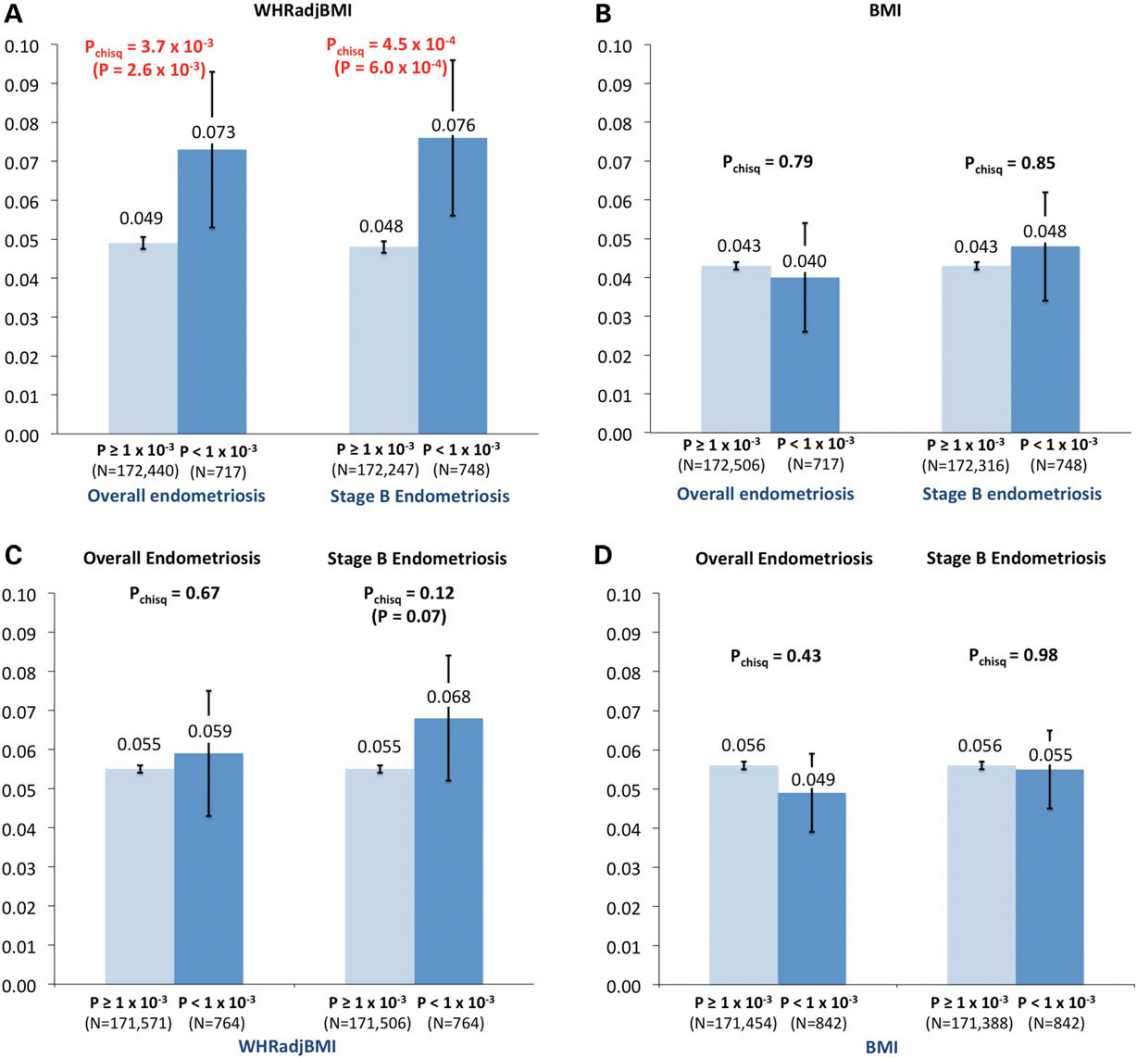
Genetic enrichment analyses between endometriosis, BMI and WHRadjBMI GWAS datasets, using independent (*r*^2^ < 0.2) SNPs. The panels show (i) The proportion of SNPs nominally associated (*P* < 0.05) with WHRadjBMI (**A**) or BMI (**B**) by significance of overall and Stage B endometriosis association (*P* < 1.0 × 10^−3^ versus *P* ≥ 1 × 10^−3^); (ii) The proportion of SNPs nominally associated (*P* < 0.05) with overall and Stage B endometriosis by significance of WHRadjBMI (C) and BMI (D) association (*P* < 1.0 × 10^−3^ versus *P* ≥ 1 × 10^−3^). *P*-values of *χ*^2^ tests assessing statistical difference between proportions are shown above each set of bars, and 95% confidence intervals of the proportions are given on each bar. For differences with *P*_chisq_ < 0.2, empirical *P*-values are given in brackets (see Supplementary Material, Methods).

To investigate whether statistical enrichment extended beyond genome-wide significant loci, we investigated the most significant (*P* < 1 × 10^−3^) independent (*r*^2^ < 0.2) endometriosis GWAS signals for enrichment of WHRadjBMI or BMI signals with *P* < 0.05 and vice versa (number of lookup SNPs per dataset: *n* = 717 to 748; see Supplementary Material, Methods). We observed statistically significant enrichment between variants associated with endometriosis (particularly Stage B) and WHRadjBMI (all endometriosis versus WHRadjBMI: *P* = 3.7 × 10^−3^; Stage B endometriosis versus WHRadjBMI: *P* = 4.5 × 10^−4^), but not between endometriosis and BMI (all endometriosis versus BMI: *P* = 0.79; Stage B endometriosis versus BMI: *P* = 0.85) (Fig. 1; Supplementary Material, Table S2). Results were similar when using female-limited WHRadjBMI (*N* = 42 969 women) and BMI (*N* = 73 137 women) GWAS summary statistics (7); to optimize power, in the remainder of the paper we therefore focus on sex-combined WHRadjBMI and BMI datasets (Supplementary Material, Fig. S1). Empirical testing of statistical enrichment through permutation (see Supplementary Material, Methods) provided near-identical results (Fig. 1; Supplementary Material, Fig. S1).

The choice of significance thresholds in the discovery and lookup datasets was based on a balance between applying a sufficiently stringent significance threshold in the discovery dataset that would minimize the proportion of false-positive association signals, while still having sufficient numbers of loci in each of the phenotypic association strata to investigate statistical enrichment, and allow the pursuit of meaningful biological pathway analyses subsequently. We considered the effect of different significance thresholds, for both discovery and lookup, which confirmed results showing enrichment of association signals between endometriosis and WHRadjBMI (Supplementary Material, Table S3), but no enrichment between endometriosis and BMI (Supplementary Material, Table S4).

To investigate potential genome-wide sharing of loci between endometriosis and WHRadjBMI or BMI, we performed polygenic prediction analyses (11) evaluating whether the aggregate effect of many variants of small effect in the WHRadjBMI and BMI GWAS could predict endometriosis status in the IEC GWAS (see Supplementary Material, Methods). There was no significant association between the WHRadjBMI- or BMI-derived profile scores (overall or female limited) and all/Stage B endometriosis (Supplementary Material, Tables S5–S8), suggesting no evidence for a directionally consistent *en masse*, genome-wide, shared common genetic component.

**Figure 2.**
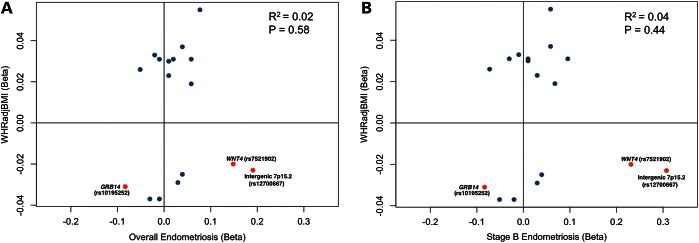
Directions of effect of 17 independent SNPs genome-wide significantly associated with all (**A**) or Stage B (**B**) endometriosis, or WHRadjBMI. Intergenic 7p15.2, *WNT4*, and *GRB14* are shown in red. Linear regression *R*^2^ and *P*-values used to test for significant directionality of effects (35) are shown.

We next investigated the variants with most significant evidence for association with both endometriosis (*P* < 1 × 10^−3^) and WHRadjBMI (*P* < 0.05) for predominance in direction of phenotypic effects (Supplementary Material, Tables S9 and S10 and Fig. S2). No statistically significant directional consistency was observed for these variants (*P* > 0.47), nor for the 17 loci (Table 1) that were genome-wide significantly associated with either trait (Fig. 2, *P* > 0.44). Intergenic 7p15.2 and *WNT4* showed discordant directions of effect, while the effect of *GRB14* was concordant (Fig. 2). This could suggest the presence of multiple biological pathways through which the variants influence the two phenotypes. We next set out to investigate the common biology suggested by genetic variants associated with both endometriosis and WHRadjBMI.

### Biology of the loci shared between endometriosis and fat distribution

Our analysis showing significant enrichment between SNPs associated with all or Stage B endometriosis (*P* < 1 × 10^−3^) and WHRadjBMI (*P* < 0.05) shown in Figure 1 involved 1284 independent (*r*^2^ > 0.2) loci. We explored the biological function of the loci most strongly associated with WHRadjBMI, at nominal *P* < 0.005 (*n* = 16, Table 2; see Supplementary Material, Tables S11 and S12 for all variants associated at *P* < 0.05). Two novel loci, rs560584 near *KIFAP3* (all endometriosis) and rs11619804 in *CAB39L* (Stage B endometriosis), were significantly associated with WHRadjBMI after Bonferroni correction allowing for 1284 independent tests (*P* < 3.89 × 10^−5^).

**Table 2.** Results of the top all/Stage B endometriosis loci (*P* < 1 × 10^−3^) associated with WHRadjBMI at *P* < 0.005

SNP	Chr	Position (B36)	RAF (allele)	Endometriosis		Overall WHRadjBMI			Female-limited WHRadjBMI			Nearest loci
				*P*-value	OR (95% CI)	*P*-value	Effect	SE	*P*-value	Effect	SE	(distance)
All cases												
rs560584	1	168 357 136	0.41 (T)	1.4 × 10^−4^	1.14 (1.07–1.22)	1.4 × 10^−5^	−0.021	0.005	1.1 × 10^−3^	−0.022	0.677	*KIFAP3* (46 632)
rs12700667	7	25 868 164	0.74 (A)	5.1 × 10^−7^	1.22 (1.13–1.32)	4.4 × 10^−5^	−0.023	0.005	3.4 × 10^−4^	−0.028	0.284	*NFE2L3* (2 90 221)
rs2921188	3	12 387 115	0.64 (A)	5.9 × 10^−4^	1.13 (1.05–1.21)	1.1 × 10^−3^	0.017	0.005	1.8 × 10^−4^	0.026	0.054	*PPARG* (0)
rs1250248	2	215 995 338	0.27 (A)	1.6 × 10^−5^	1.17 (1.09–1.26)	1.0 × 10^−3^	0.018	0.005	9.9 × 10^−4^	0.025	0.242	*FN1* (0)
rs2630787	3	21 847 339	0.52 (C)	9.2 × 10^−4^	1.12 (1.05–1.19)	1.9 × 10^−3^	−0.015	0.004	0.38	−0.006	0.030	*ZNF659* (79 518)
rs1430788	2	67 721 916	0.31 (C)	9.3 × 10^−5^	1.15 (1.07–1.23)	2.7 × 10^−3^	0.016	0.005	3.1 × 10^−3^	0.022	0.330	*ETAA1* (230 878)
rs906721	3	184 687 691	0.41 (A)	6.1 × 10^−5^	1.16 (1.08–1.24)	4.2 × 10^−3^	0.015	0.005	1.7 × 10^−3^	0.023	0.140	*KLHL6* (322)
rs1868894	4	187 606 728	0.80 (C)	2.3 × 10^−4^	1.16 (1.07–1.26)	4.9 × 10^−3^	−0.018	0.006	0.13	−0.013	0.524	*MTNR1A* (85 075)
rs3820282	1	22 340 802	0.16 (T)	3.3 × 10^−7^	1.26 (1.15–1.37)	5.0 × 10^−3^	−0.019	0.007	0.09	−0.016	0.749	*WNT4* (0)
Stage B cases												
rs11619804	13	49 888 131	0.53 (C)	4.8 × 10^−4^	1.17 (1.07–1.28)	1.1 × 10^−5^	0.022	0.005	2.2 × 10^−2^	0.016	0.022	*CAB39L* (0)
rs12700667	7	25 868 164	0.74 (A)	3.3 × 10^−9^	1.36 (1.23–1.50)	4.4 × 10^−5^	−0.023	0.005	3.4 × 10^−4^	−0.028	0.284	*NFE2L3* (290 221)
rs2782659	6	45 794 768	0.33 (G)	4.2 × 10^−4^	1.18 (1.08–1.30)	9.2 × 10^−5^	0.020	0.005	1.7 × 10^−4^	0.027	0.108	*RUNX2* (167 970)
rs6556301	5	176 460 183	0.63 (G)	7.4 × 10^−4^	1.17 (1.07–1.28)	1.9 × 10^−4^	−0.021	0.005	7.8 × 10^−3^	−0.021	0.845	*FGFR4* (2450)
rs1250248	2	215 995 338	0.27 (A)	2.9 × 10^−8^	1.32 (1.19–1.45)	1.2 × 10^−3^	0.018	0.005	9.9 × 10^−4^	0.025	0.242	*FN1* (0)
rs4131816	1	161 662 648	0.85 (T)	5.4 × 10^−4^	1.24 (1.10–1.41)	1.5 × 10^−3^	0.022	0.007	0.25	0.011	0.072	*NUF2* (70 470)
rs9912335	17	77 552 948	0.69 (T)	3.1 × 10^−4^	1.19 (1.08–1.31)	3.5 × 10^−3^	−0.021	0.007	0.10	−0.016	0.454	*ASPSCR1* (0)
rs10878362	12	64 703 760	0.69 (C)	4.9 × 10^−4^	1.19 (1.08–1.31)	3.6 × 10^−3^	0.015	0.005	3.1 × 10^−3^	0.022	0.204	*HMGA2* (57 421)
rs2807357	1	22 364 571	0.64 (A)	9.7 × 10^−4^	1.16 (1.06–1.27)	3.7 × 10^−3^	−0.015	0.005	1.0 × 10^−3^	−0.024	0.081	*WNT4* (22 373)
rs906721	3	184 687 691	0.41 (A)	1.4 × 10^−4^	1.20 (1.09–1.32)	4.2 × 10^−3^	0.015	0.005	1.7 × 10^−3^	0.023	0.140	*KLHL6* (322)
rs12267660	10	4 419 530	0.85 (G)	7.9 × 10^−4^	1.24 (1.09–1.40)	4.6 × 10^−3^	0.02	0.007	8.0 × 10^−3^	0.030	0.133	*CR749391* (191 913)
rs11685481	2	67 590 253	0.15 (C)	8.4 × 10^−4^	1.23 (1.09–1.38)	4.8 × 10^−3^	0.018	0.006	1.1 × 10^−2^	0.022	0.451	*ETAA1* (99 215)

The endometriosis risk allele T of rs560584 (OR = 1.14 (1.07–1.22), *P* = 1.42 × 10^−4^) was associated with lower WHRadjBMI (*β* = −0.021, *P* = 1.47 × 10^−5^), and located in an intergenic region 46 kb downstream of *KIFAP3* (*Kinesin-associated protein 3*). Together with *KIF3A* and *KIF3B*, *KIFAP3* forms a kinesin motor complex, KIF3, that mediates cellular transport of N-cadherin and β-catenins (12), which are involved in cell adhesion, the *Wnt* canonical pathway and cell cycle progression (13). The *Wnt/β*-catenin signalling pathway acts as a molecular switch for adipogenesis (14) and has multiple suggested roles in endometriosis through sex hormone homeostasis regulation (15), its role in development of female reproductive organs (16), molecular mechanisms of infertility (17) and mediation of fibrogenesis (18).

The Stage B endometriosis risk allele C of rs11619804 (OR = 1.17 (1.07–1.28); *P* = 4.88 × 10^−4^), located in *CAB39L* (Calcium-Binding Protein 39-Like), was associated with increased WHRadjBMI (*β* = 0.022, *P* = 1.06 × 10^−5^; Table 2). The function of this gene is not well characterized but the encoded protein interacts with a serine threonine kinase (*STK11*) that functions as a tumour suppressor (19).

Rs12700667 in the intergenic region 7p15.2 remained among the strongest associated shared signals, with the endometriosis risk allele A associated with reduced WHRadjBMI (*β* = −0.023, *P* = 4.4 × 10^−5^). The association maps to an intergenic high LD region of 48 kb (*r*^2^ > 0.8) of unknown functionality. Further interesting nearby loci include the miRNA *hsa-mir-148a*, with a purported role in *Wnt/β*-catenin signalling (14); *NFE2L3* (nuclear factor (erythroid-derived 2)-like 3), a transcription factor suggested to be involved in cell differentiation, inflammation and carcinogenesis (20). The *WNT* signalling pathway was further highlighted by the nominal association of two independent (*r*^2^ = 0.06) endometriosis risk variants near *WNT4* (wingless-type MMTV integration site family), rs3820282 (genic) and rs2807357 (22.4 kb downstream), with reduced WHRadjBMI (*β* = −0.019, *P* = 5.0 × 10^−3^; *β* = −0.015, *P* = 3.7 × 10^−3^; Table 2). Of note is that all shared variants implicated in *WNT* signalling (in/near intergenic 7p15.2, *WNT4*, *KIFAP3*) showed consistent—discordant—phenotypic directions of effect.

Risk variant rs10195252, 34.6 kb downstream of *GRB14* (growth factor receptor-bound protein 14) was the third locus with significant evidence for association with both overall (not Stage B) endometriosis and WHRadjBMI (Table 1). *GRB14* has an inhibitory effect on insulin receptor signalling (21), may have a role in signalling pathways that regulate growth and metabolism and has been shown to interact with fibroblast growth factor receptors (22). This shared variant is also in high LD (*r*^2^ = 0.93 and = 0.87, respectively) with a type 2 diabetes risk variant rs13389219 (23) and fasting insulin risk variant rs6717858 (24).

Other loci of interest include rs2921188 in *PPARG* and rs6556301 near *FGFR4* (Table 2)*.* The endometriosis risk allele A of rs2921188 (OR = 1.13, 95% CI: 1.05–1.21), *P* = 5.9 × 10^−4^) in *PPARG* (peroxisome proliferator-activated receptor gamma) is associated with increased WHRadjBMI (*β* = 0.017; *P* = 1.1 × 10^−3^). *PPARG* is a nuclear hormone receptor that regulates fatty acid storage and glucose metabolism. Synthetic ligands, such as insulin sensitizing drugs, target *PPARG* in treatment of diabetes to lower serum glucose levels (25) and are also documented to have anti-inflammatory, anti-angiogenic and anti-proliferative effects on endometrium, with baboon models suggesting a role in targeting endometriotic disease (26). Stage B endometriosis risk allele G of rs6556301 near *FGFR4* (*fibroblast growth factor receptor*, OR = 1.17 [1.07–1.28], *P* = 7.4 × 10^−4^) is associated with reduced WHRadjBMI (*β* = −0.021, *P* = 1.9 × 10^−4^). *FGFR4* interacts with fibroblast growth factors, which have roles in angiogenesis, wound healing and cell migration (27).

### Expression quantitative trait loci analysis of the shared endometriosis and fat distribution loci

We investigated the potential impact of the described 16 genes (Table 2) shared between endometriosis and WHRadjBMI on transcriptional function using three public expression data resources: (i) the Mammalian Gene Expression Uterus database (MGEx-Udb) (28) containing published information on transcriptional activity of specific genes in human endometrial tissue from individuals with and without endometriosis; (ii) the MuTHER study which collected expression and eQTL data from 776 abdominal fat tissues (29); and (iii) the MOLOBB dataset of differential expression levels between abdominal and gluteal fat from 49 individuals (30). Based on the limited available evidence in the MGEx-Udb database, two genes are transcribed in endometrial tissue of women with endometriosis but dormant in those without endometriosis: *PPARG* and *FGFR4* (Supplementary Material, Table S13). Of the 16 genes, 15 had probes present within 1 Mb either side of the SNP in the MuTHER database; however, none showed significant association with nearby transcripts in abdominal fat tissue (Supplementary Material, Table S14). The MOLOBB study data showed *cis*-eQTL evidence for differential expression of two genes; *KIFAP3* (rs560584; fold change = 0.14, adjusted *P* = 0.04) (Supplementary Material, Table S15). Additional transcriptional evidence relevant to the intergenic 7p15.2 locus includes the presence of an expression QTL associated with a transcript of unknown function, *AA553656*, in subcutaneous abdominal fat tissue (6), and the differential expression of nearby *hsa-miR-148a* between gluteal and abdominal fat tissue samples (31).

### Pathway analysis

To identify potential common biological pathways involved in the aetiology of endometriosis and the variability of fat distribution, we conducted pathway analyses using genes with evidence for enrichment between the traits using (i) the PANTHER database (32) and (ii) GRAIL (33). For the PANTHER analysis, we selected the 91 and 108 genes located in a 1 Mb interval surrounding each independent SNP associated with all endometriosis (*P* < 1.0 × 10^−3^) and WHRadjBMI (*P* < 0.05), and Stage B endometriosis (*P* < 1.0 × 10^−3^) and WHRadjBMI (*P* < 0.05), respectively (see Supplementary Material, Methods). This excluded intergenic loci without a gene within 1 Mb, such as our top shared locus at 7p15.2. We tested whether the two sets of genes showed significant overrepresentation of a particular pathway, for each of 176 curated pathways and 241 biological processes. The top enriched pathways were ‘developmental processes’ (all endometriosis: *P* = 1.2 × 10^−5^; Stage B: *P* = 1.25 × 10^−4^), ‘*WNT* signalling’ (all endometriosis: *P* = 1.07 × 10^−4^), ‘gonadotropin-releasing hormone receptor’ (all endometriosis: *P* = 1.48 × 10^−3^), ‘cadherin signalling’ (Stage B: *P* = 6.42 × 10^−4^), ‘FGF signalling’ (Stage B: *P* = 2.96 × 10^−3^) and ‘TGF-beta signalling’ (Stage B: *P* = 1.48 × 10^−3^) pathways (Supplementary Material, Tables S16 and S17). Bonferroni correction for the number of pathways tested (see Supplementary Material, Methods) rendered ‘*WNT* signalling’, ‘developmental processes’, ‘cellular processes’ and ‘cell communication’ significantly enriched; however, this adjustment is conservative, as exemplified by ‘cadherin signalling’ genes being a subset of those in the ‘*WNT* signalling’ pathway. Sensitivity analyses exploring the effect of different endometriosis association thresholds on pathway analyses showed very consistent results for threshold *P* < 1.0 × 10^−2^, with the same top three enriched pathways—*WNT* signalling, Cadherin signalling and Gonadotropin-releasing hormone receptor pathway. No meaningful pathway analyses could be conducted on the limited number of genes passing association threshold *P* < 1 × 10^−4^ (Supplementary Material, Table S18).

We used GRAIL (33) to search for connectivity between the 91 and 108 genes all/Stage B endometriosis and WHRadjBMI-associated genes and specific keywords from the published literature that describe potential functional connections. We identified 17 genes with nominal significance (*P* < 0.05) for potential functional connectivity for ‘all’ endometriosis and WHRadjBMI and six genes for Stage B endometriosis and WHRadjBMI (Supplementary Material, Fig. S3 and Tables S19 and S20). The keywords associated with these connections included ‘cadherin’, ‘differentiation’, ‘development’ and ‘insulin’ for ‘all’ endo, and ‘development’ and ‘embryos’ for Stage B endometriosis, marking again developmental processes and cadherin signalling as biological pathways shared in the origins of endometriosis and fat distribution.

## DISCUSSION

In this study, we have investigated the overlap in genetic association signals from the largest GWA studies to date of endometriosis, overall adiposity (BMI) and fat distribution (WHRadjBMI). Our results demonstrated that there is a shared genetic basis between endometriosis and fat distribution that extends over and above the single genome-wide significant locus that has been reported in GWAS of the separate traits. Our analyses highlight novel loci in/near *KIFAP3* and *CAB39L*, which together with intergenic 7p15.2, *WNT4* and *GRB14*, showed significant evidence of trait association sharing. The strength of evidence of enrichment was similar for overall versus female-limited WHRadjBMI loci, which may be unexpected, given that endometriosis is a female condition. However, the lack of a stronger enrichment between female-specific WHRadjBMI GWAS results and endometriosis, compared with all WHRadjBMI results should be considered against the effects of a reduced sample size used for female-specific WHRadjBMI analyses on power of association detection.

The enrichment of associated variants was generally stronger when the endometriosis cases were restricted to moderate–severe (Stage B) disease, despite the smaller sample size. Indeed, the association of the top intergenic GWAS locus on 7p15.2, also genome-wide significantly associated with WHRadjBMI, is limited to Stage B endometriosis. Stage B—or ASRM Stages III/IV disease (34)—is typically characterized by ovarian (endometrioma) or deep infiltrating (rectovaginal) lesions, which were shown to have a substantially greater underlying genetic contribution than milder, peritoneal disease (ASRM Stage I/II) (3). The particular enrichment between WHRadjBMI and Stages III/IV endometriosis is intriguing, and another reason for further functional work to concentrate on this endometriosis sub-type. There are, however, specific loci that show enrichment of association with WHRadjBMI and overall endometriosis, the analysis of which therefore remains of interest. An example is *GRB14*, which did not show significant association with Stage B disease, displayed a concordant direction of effect between endometriosis and WHRadjBMI, and the biological function of which also seems to suggest an entirely different contribution to the origins of both phenotypes than the 7p15.2 and *WNT4* loci.

The limited available eQTL data showed significant evidence for differential expression of *KIFAP3* between different fat depots. The variants with most evidence for enrichment between the traits, in/near intergenic 7p15.2, *KIFAP3* and *WNT4*, were all implicated in *WNT* signalling and had consistent—discordant—directions of effect, with endometriosis risk alleles associated with a decreased WHRadjBMI. Indeed, biological pathway analyses showed significant evidence for the involvement of developmental processes and *WNT* signalling in endometriosis aetiology and regulation of fat distribution, a potential pleiotropic connection that has not been reported to date.

The relatively limited epidemiological evidence of phenotypic correlation between endometriosis and WHRadjBMI (8,9) is consistent with the absence of strong directional consistency of phenotypic effects of genetic variants underlying both traits at a genome-wide level. Most studies of genetic pleiotropy between traits to date have focused on genome-wide directional consistency between epidemiologically or clinically (postulated) correlated traits, such as different metabolic traits (6,35) or psychiatric conditions (36). However, genome-wide consistency in directionality of phenotypic effects would most likely apply to traits that share a large proportion of causality, and that epidemiologically lie on the same causal pathway(s) and are thus more likely to be examples of mediated (genetic variants influencing one phenotype indirectly through association with a second phenotype) rather than biological (genetic variants exerting a direct biological influence on more than one phenotype) pleiotropy (37). Thus, our results of genetic enrichment between endometriosis and WHRadjBMI demonstrate an example of the biological complexity of aetiological associations between complex traits, and suggest that the underlying shared loci are potentially biologically pleiotropic, given the absence of phenotypic correlation between endometriosis and WHRadjBMI and absence of *en masse* directional consistency of shared genetic variants on the phenotypes (37,38). It also demonstrates more generally how potential perturbation of a causal pathway through, for example, drug treatment targeting one trait could have unexpected effects on another, even when there is no clear evidence that these traits are associated clinically or epidemiologically—a problem often encountered in drug development. Systematic exploration of biological pleiotropy of genetic variants marking potential drug targets may help in highlighting the potential of such unwanted or unexpected effects.

While the observed genetic enrichment between endometriosis and WHRadjBMI presents new avenues for exploring common biology, the total absence of any genetic enrichment between endometriosis and BMI (within the limits of power presented by these large datasets) is intriguing given the consistent, prospective, observational epidemiological evidence of phenotypic association between reduced BMI and endometriosis risk (8). Our analyses represent an adaptation of Mendelian randomization analyses (39,40), in which genetic variants robustly associated with BMI in the largest GWAS analyses to date (10) are investigated for association with endometriosis. The total lack of genetic enrichment suggests that reduced BMI is not causally related to endometriosis risk. Rather, it suggests that the observed phenotypic association (8) is either driven by shared environmental factors, or is due to confounding factors related to BMI affecting, for example, diagnostic opportunity for endometriosis.

These novel findings present an entirely new opportunity for functional targeted follow-up of pleiotropic loci between endometriosis and WHRadjBMI in relevant disease tissues such as endometrium and fat tissue, cellular systems, animal models and further cross-trait comparisons, to uncover their biological functions and to assess how studies in the fat distribution research field can inform research into endometriosis pathogenesis, biomarker identification and drug target discovery and validation.

## MATERIALS AND METHODS

### Genome-wide association studies

#### IEC endometriosis GWAS

This GWAS included 3194 surgically confirmed endometriosis cases and 7060 controls from Australia and the UK. Disease severity of the endometriosis cases was assessed retrospectively from surgical records using the rAFS classification system and grouped into two phenotypes: Stage A (Stage I or II disease or some ovarian disease with a few adhesions; *n* = 1686) or Stage B (Stage III or IV disease; *n* = 1364). We previously showed an increased genetic loading among 1364 cases with Stage B endometriosis compared with 1666 with Stage A disease (3), which led to two GWA analyses, including (i) 3194 ‘all’ endometriosis case and (ii) 1364 Stage B cases (Table 3). The genotyped data were imputed up to 1000 Genomes pilot reference panel (B36, June 2010) and the GWAS was performed again, using a missing data likelihood in a logistic regression model including a covariate representing the Australian and the UK strata, with the imputed data (*N* = 12.5 million SNPs). The enrichment analysis we present is from this set of results.

**Table 3.** Summary description of the GWAS used in the genetic enrichment analysis

GWAS	Consortium	Sample size	No. of SNPs (million)	References
Endometriosis—all cases	IEC	3194 cases, 7060 controls	∼12.5	Painter *et al*. (3)
Endometriosis—Stage B cases	IEC	1363 cases, 7060 controls	∼12.5	Painter *et al*. (3)
WHRadjBMI	GIANT	77 167	∼2.85	Heid *et al*. (6)
Female-limited WHRadjBMI	GIANT	42 969	∼2.85	Randall *et al*. (7)
BMI	GIANT	123 865	∼2.85	Speliotes *et al*. (10)
Female-limited BMI	GIANT	73 137	∼2.85	Randall *et al*. (7)

IEC, International Endogene Consortium; GIANT, Genetic Investigation of Anthropometric Traits Consortium; BMI, body mass index adjusted for age; WHRadjBMI, waist to hip ratio adjusted for BMI and age.

#### GIANT Consortium

##### WHR GWAS

A total of 77 167 subjects of European ancestry informative of body fat distribution measurement WHR from 32 GWAS were included (6). The genotype data were imputed up to HapMap 2 CEU reference panel. The associations of 2.85 million SNPs with WHR were examined in a fixed-effects meta-analysis, after inverse normal transformation of WHR and adjusting for BMI and age within each study in an additive genetic model; analyses were conducted for males and females combined (6) and limited to females only (7) (Table 3).

##### BMI GWAS

A total of 123 865 subjects with overall adiposity measurement BMI from 46 GWAS were included (10). The genotype data were imputed up to HapMap two CEU reference panels. The associations of 2.85 million SNPs with BMI were tested in an inverse-variance meta-analysis, after inverse normally transformation of BMI and adjusting for age and other appropriate covariates in an additive genetic model within each study; analyses were conducted for males and females combined (10) and limited to females only (7) (Table 3).

### Genetic enrichment analysis

With one test of association conducted for each SNP, the GWAS analyses produced a genome-wide distribution of *P*-values of individual SNP associations. Prior to testing enrichment: (i) the overlap of SNPs present in endometriosis GWAS versus WHRadjBMI and BMI GWAS was taken, (ii) all SNPs with MAF ≤ 0.01 were removed, (iii) all SNPs with A/T and C/G base pairs were removed, (iv) correlated SNPs (*r*^2^ > 0.2) were removed as previously reported (41) by taking the most significantly associated SNP and eliminating all SNPs that have a HapMap CEU pairwise correlation coefficient (*r*^2^) > 0.2 with that SNP, then processing to the next strongly associated SNP remaining. This resulted in 173 157 independent SNPs in endometriosis versus WHRadjBMI and 173 223 in endometriosis versus BMI enrichment analyses.

The independent SNPs in the tails (*P* < 1 × 10^−3^) of the association results distribution of the two endometriosis GWAS (all endometriosis and ‘Stage B’ cases) were investigated for enrichment of WHRadjBMI or BMI low *P*-value (*P* < 0.05) association signals; in reversal, SNPs in the tails of WHRadjBMI and BMI GWAS (*P* < 1 × 10^−3^) were investigated for evidence of nominal association (*P* < 0.05) in the two endometriosis GWAS. The threshold of *P* < 1 × 10^−3^ corresponded to the point at which endometriosis GWAS results started to deviate from the null distribution (evidence for association) in the overall and Stage B endometriosis Q–Q plots (Supplementary Material, Fig. S4). Enrichment was assessed in R by means of Pearson's *χ*^2^ tests with Yates' continuity correction, testing for the difference in proportion of SNPs with association *P* < 0.05 in the lookup dataset according to association in the discovery dataset (*P* < 1 × 10^−3^ versus *P* ≥ 1 × 10^−3^). To test for consistency in directionality of phenotypic effects of the SNPs with evidence of enrichment, linear regression analysis was performed on the effect (*β*) of each SNP for WHRadjBMI as predictor variable and the effect (*β*) of endometriosis risk as the outcome variable (35). In addition, a two-sided binomial test was performed with null hypothesis *P* = 0.50.

### Permutation-based enrichment analysis

For those results that showed nominally significant (*P* < 0.10) evidence for enrichment in *χ*^2^ tests of contingency tables, we performed permutation-based analyses to obtain empirical estimates of significance of enrichment. We (i) randomly picked the same number of independent SNPs ‘associated’ with the discovery trait at *P* < 1 × 10^−3^ (e.g. the number of SNPs associated with all endometriosis at *P* < 1 × 10^−3^ was *n* = 717) from the WHRadjBMI dataset; (ii) counted how many of the randomly selected SNPs had *P*-values of association with WHRadjBMI <0.05; (iii) repeated Steps (i) and (ii) 10 000 times; (iv) determined the number of instances among the 10 000 draws in which the number of SNPs associated at *P* < 0.05 with WHRadjBMI was greater or equal to the number we observed in our original analysis (e.g. ≥52/717). For example, for overall endometriosis and overall WHRadjBMI, we observed this in 26/10 000 instances, corresponding to a *P*-value of 2.6 × 10^−3^, which was very similar to the *P*-value obtained from the *χ*^2^ test (*P* = 3.7 × 10^−3^).

### Polygenic prediction analysis

The independent SNPs in both WHRadjBMI and endometriosis datasets were used to conduct a polygenic prediction analysis (11). The aim of this analysis was to evaluate the aggregate effects of many SNPs of small effect and assess whether subsets of SNPs selected in this manner from one disease/trait GWAS predict disease/trait status in another, thus providing a measure of a common polygenic component with concordant directions of effect underlying the traits. Briefly, subsets of SNPs were selected from the WHRadjBMI GWAS data based on their association with WHRadjBMI using increasingly liberal thresholds, that is, *P* < 0.01, *P* < 0.05, *P* < 0.1, *P* < 0.2, *P* < 0.3, *P* < 0.4, *P* < 0.5 and *P* < 0.75. Using these thresholds, we defined sets of allele-specific scores in the WHRadjBMI dataset to generate risk profile scores for individuals in the endometriosis dataset. For each individual in the endometriosis dataset, we calculated the number of score alleles they possessed, each weighted by their effect size (*β*-value) of association in the WHRadjBMI dataset. To assess whether the aggregate scores were associated with endometriosis risk, we tested for a higher mean score in cases compared with controls. Logistic regression was used to assess the relationship between endometriosis disease status and aggregate risk score.

### Expression analyses

#### MGEx-Udb

The mammalian gene expression uterus database (MGEx-Udb) is a manually curated uterus-specific database created using a meta-analysis approach from published papers (28) that provides lists of transcribed and dormant genes for various normal, pathological (e.g. endometriosis, cervical cancer and endometrial cancer) and experimental (e.g. treatment and knockout) conditions. Each gene's expression status is indicated by a reliability score, derived based on the consensus across multiple samples and studies which highly variable (http://resource.ibab.ac.in/MGEx-Udb/).

#### MuTHER

The MuTHER resource includes LCLs, skin and adipose tissue-derived simultaneously from a subset of well-phenotyped healthy female twins (29). Whole-genome expression profiling of the samples, each with either two or three technical replicates, was performed using the Illumina Human HT-12 V3 BeadChips (Illumina, Inc.) according to the protocol supplied by the manufacturer. Log2 transformed expression signals were normalized separately per tissue as follows: quantile normalization was performed across technical replicates of each individual followed by quantile normalization across all individuals.

Genotyping was conducted using a combination of Illumina arrays (HumanHap300, HumanHap610Q, 1M-Duo and 1.2MDuo 1 M). Untyped HapMap2 SNPs were imputed using the IMPUTE software package (v2). In total, there were 776 samples with genotypes and expression values in adipose tissue. Association between all SNPs (MAF > 5%, IMPUTE info score > 0.8) within a gene or within 1 Mb of the gene transcription start or end site, and normalized expression values, were performed with the GenABEL/ProbABEL packages (42) using polygenic linear models incorporating a kinship matrix (GenABEL) followed by the mm score test with imputed genotypes (ProbABEL). Age and experimental batch were included as cofactors in the analysis. Benjamini Hochberg corrected *P*-values are reported.

#### MolOBB

We performed differential *cis*-eQTL analysis to compare the expression levels in gluteal and abdominal fat tissue from 49 individuals in the MolOBB dataset (24 with and 25 without metabolic syndrome—MetSyn) (30). We first checked for the presence of the SNP in the MolOBB genotype data and, in the case of absence, selected any proxies (*r*^2^ > 0.8) available. We then searched for nearby genes (±500 kb) covered by the expression data using the bioconductor R package GenomicRanges (43) and tested for association at each pair using a linear model with the expression level as an outcome and the SNP allelic dosage as a predictor, adjusting for age, gender and MetSyn case–control status. This analysis was carried out for both abdominal and gluteal subcutaneous adipose tissue. To investigate whether genes were differentially expressed between the two tissues, we applied a linear mixed model with tissue, MetSyn case–control status, gender and plate were as fixed effects, and subject as a random effect using MAANOVA (44), as previously described in Min *et al*. (30). We report the uncorrected and genome-wide FDR corrected *F*s test *P*-values (30).

### Biological pathway analysis

#### PANTHER

We conducted analyses using the PANTHER 8.1 database containing pathway information on 20 000 genes (*Homo sapiens*) (32). We selected independent SNPs, which had association *P*-values < 1 × 10^−3^ in the endometriosis datasets and an association *P*-value of <0.05 in the WHRadjBMI dataset, resulting in (i) 91 SNPs for all endometriosis and WHRadjBMI and (ii) 108 SNPs for Stage B endometriosis and WHRadjBMI. Each SNP was mapped to the closest gene within 1 Mb; 88 of 91 and 103 of 108 genes were present in the PANTHER database, and these subsets were tested for correlation with 241 biological processes and 176 pathways classified in the database (32). For each biological process/pathway, the difference between the observed fraction of genes in that pathway and the number expected by chance was tested using Fisher exact test. A Bonferroni correction was used as a conservative method for adjusting for the maximum number of biological processes (*n* = 278; *P* = 1.80 × 10^−4^) and pathways (*n* = 78; *P* = 6.41 × 10^−4^) tested.

## SUPPLEMENTARY MATERIAL

Supplementary Material is available at *HMG* online.

## FUNDING

The endometriosis GWAS was supported by a grant from the Wellcome Trust (WT084766/Z/08/Z) and makes use of WTCCC2 control data generated by the Wellcome Trust Case-Control Consortium. A full list of the investigators who contributed to the generation of these data is available from http://www.wtccc.org.uk. Funding for the WTCCC project was provided by the Wellcome Trust under awards 076113 and 085475. The QIMR study was supported by grants from the National Health and Medical Research Council (NHMRC) of Australia (241944, 339462, 389927, 389875, 389891, 389892, 389938, 443036, 442915, 442981, 496610, 496739, 552485 and 552498), the Cooperative Research Centre for Discovery of Genes for Common Human Diseases (CRC), Cerylid Biosciences (Melbourne) and donations from N. Hawkins and S. Hawkins. S.M. was supported by NHMRC Career Development Awards (496674, 613705). D.R.N. was supported by the NHMRC Fellowship (339462 and 613674) and the ARC Future Fellowship (FT0991022) schemes. A.P.M. was supported by a Wellcome Trust Senior Research Fellowship. G.W.M. was supported by the NHMRC Fellowships Scheme (339446, 619667). K.T.Z. was supported by a Wellcome Trust Research Career Development Fellowship (WT085235/Z/08/Z). C.M.L. was supported by a Wellcome Trust Research Career Development Fellow (086596/Z/08/Z). N.R. was supported by an MRC grant (MR/K011480/1). Funding to pay the Open Access publication charges for this article was provided by the Wellcome Trust.

## Supplementary Material

Supplementary Data

## APPENDIX

### The International Endogene Consortium

Carl A. Anderson^1,2^, Scott D. Gordon^3^, Qun Guo^4^, Anjali K. Henders^3^, Ann Lambert^5^, Sang Hong Lee^6^, Peter Kraft^7^, Stephen H. Kennedy^5^, Stuart Macgregor^3^, Nicholas G. Martin^3^, Stacey A. Missmer^4^, Grant W. Montgomery^3^, Andrew P. Morris^1^, Dale R. Nyholt^3^, Jodie N. Painter^3^, Fenella Roseman^5^, Susan A. Treloar^8^, Peter M. Visscher^9^, Leanne Wallace^3^, Krina T. Zondervan^1,5^.

^1^Wellcome Trust Centre for Human Genetics, University of Oxford, Oxford, UK, ^2^Wellcome Trust Sanger Institute, Wellcome Trust Genome Campus, Hinxton, UK, ^3^Queensland Institute of Medical Research, Herston, QLD, Australia, ^4^Brigham and Women's Hospital and Harvard Medical School, Boston, MA, USA, ^5^Nuffield Department of Obstetrics and Gynaecology, University of Oxford, John Radcliffe Hospital, Oxford, UK, ^6^Queensland Brain Institute, The University of Queensland, Brisbane, QLD 4072, Australia, ^7^Harvard School of Public Health, Boston, MA, USA, ^8^Centre for Military and Veterans' Health, The University of Queensland, Mayne Medical School, QLD, Australia, ^9^The University of Queensland Diamantina Institute, Princess Alexandra Hospital, Brisbane, QLD 4102, Australia.

### The GIANT Consortium

Joshua C. Randall^1,2^, Thomas W. Winkler^3^, Zoltan Kutalik^4,5^, Sonja I. Berndt^6^, Anne U. Jackson^7^, Keri L. Monda^8^, Tuomas O. Kilpelainen^9^, Tonu Esko^10,11^, Reedik Magi^2,10^, Shengxu Li^9,12^, Tsegaselassie Workalemahu^13^, Mary F. Feitosa^14^, Damien C. Croteau-Chonka^15^, Felix R. Day^9^, Tove Fall^16^, Teresa Ferreira^2^, Stefan Gustafsson^16^, Adam E. Locke^7^, Iain Mathieson^2^, Andre Scherag^17^, Sailaja Vedantam^18,19,20^, Andrew R. Wood^21^, Liming Liang^22,23^, Valgerdur Steinthorsdottir^24^, Gudmar Thorleifsson^24^, Emmanouil T. Dermitzakis^25^, Antigone S. Dimas^2,25,26^, Fredrik Karpe^27^, Josine L. Min^2^, George Nicholson^28,29^, Deborah J. Clegg^30^, Thomas Person^30^, Jon P. Krohn^2^, Sabrina Bauer^31^, Christa Buechler^31^, Kristina Eisinger^31^, DIAGRAM Consortium, Amelie Bonnefond^32^, Philippe Froguel^32,33^, MAGIC Investigators, Jouke-Jan Hottenga^34^, Inga Prokopenko^2,27^,Lindsay L. Waite^35^, Tamara B. Harris^36^, Albert Vernon Smith^37,38^, Alan R. Shuldiner^39,40^, Wendy L. McArdle^41^, Mark J. Caulfield^42^, Patricia B. Munroe^42^, Henrik Gonberg^16^, Yii-Der Ida Chen^43,44^, Guo Li^45^, Jacques S. Beckmann^46,4^, Toby Johnson^4,5,42^, Unnur Thorsteinsdottir2^4,47^, Maris Teder-Laving^10^, Kay-Tee Khaw^48^, Nicholas J. Wareham^9^, Jing Hua Zhao^9^, Najaf Amin4^9^,Ben A. Oostra5^0,51,52^, Aldi T. Kraja1^4^, Michael A. Province^14^, L. Adrienne Cupples^53^, Nancy L. Heard- Costa^54^, Jaakko Kaprio^55,56,57^, Samuli Ripatti^1,57,58^, Ida Surakka^57,58^, Francis S. Collins^59^, Jouko Saramies^60^, Jaakko Tuomilehto^61,62,63,64^, Antti Jula^65^, Veikko Salomaa^66^,Jeanette Erdmann^67,68^, Christian Hengstenberg^69^, Christina Loley^68,70^, Heribert Schunkert^70^, Claudia Lamina^71^, H. Erich Wichmann^72,73^, Eva Albrecht^74^, Christian Gieger^74^, Andrew A. Hicks^75^, Asa Johansson^76,77^, Peter P. Pramstaller^75,78,79^, Sekar Kathiresan8^0,81,82^, Elizabeth K. Speliotes^83,84^, Brenda Penninx^85^, Anna-Liisa Hartikainen^86^, Marjo-Riitta Jarvelin^87,88,89,90^, Ulf Gyllensten^76^,Dorret I. Boomsma^34^, Harry Campbell^91^, James F. Wilson^91^, Stephen J. Chanock^6^, Martin Farrall^92^, Anuj Goel9^2^, Carolina Medina-Gomez^49,52,93^, Fernando Rivadeneira^49,52,93^, Karol Estrada^49,52,93^, Andre G. Uitterlinden4^9,52,93^, Albert Hofman^49,52^, M. Carola Zillikens^52,93^, Martin den Heijer^94^, Lambertus A. Kiemeney^95,96,97^, Andrea Maschio^98^, Per Hall^16^, Jonathan Tyrer^99^, Alexander Teumer^100^, Henry Volzke^101^, Peter Kovacs^102^, Anke Tonjes^103,104^, Massimo Mangino^105^, Tim D. Spector^105^, Caroline Hayward1^06^, Igor Rudan^91^, Alistair S. Hall^107^, Nilesh J. Samani^108,109^, Antony Paul Attwood1^,110^, Jennifer G. Sambrook^110,111^, Joseph Hung^112,113^, Lyle J. Palmer^114,115^, Marja- Liisa Lokki^116^, Juha Sinisalo^117^, Gabrielle Boucher^118^, Heikki Huikuri^119^, Mattias Lorentzon^120^, Claes Ohlsson^120^, Niina Eklund^11,58^, Johan G. Eriksson^121,122,123^, Cristina Barlassina^124^, Carlo Rivolta^4^, Ilja M. Nolte^125^, Harold Snieder^125,126^, Melanie M. Van der Klauw^126,127^, Jana V. Van Vliet-Ostaptchouk1^26,127^, Pablo V. Gejman^128,129^, Jianxin Shi^6^, Kevin B. Jacobs^6,130^, Zhaoming Wang^6,130^, Stephan J. L. Bakker^131^, Irene Mateo Leach^132^, Gerjan Navis^131^, Pim van der Harst^132,133^, Nicholas G. Martin^134^, Sarah E. Medland^134^, Grant W. Montgomery^135^, Jian Yang^136^, Daniel I. Chasman^137,138^, Paul M. Ridker^137,138^, Lynda M. Rose^137^, Terho Lehtimaki^139^,Olli Raitakari^140,141^, Devin Absher^35^, Carlos Iribarren^142^, Hanneke Basart^143^, Kees G. Hovingh^143^, Elina Hypponen^144^, Chris Power^144^, Denise Anderson1^45,146^, John P. Beilby^113,147,148^, Jennie Hui^113,147,148,149^, Jennifer Jolley^110^, Hendrik Sager^150^, Stefan R. Bornstein^151^,Peter E. H. Schwarz^151^, Kati Kristiansson^57,58^, Markus Perola^10,57,58^, Jaana Lindstrom^63^, Amy J. Swift^59^, Matti Uusitupa1^52,153^, Mustafa Atalay^154^, Timo A. Lakka1^54,155^, Rainer Rauramaa^155,156^, Jennifer L. Bolton^91^, Gerry Fowkes^91^, Ross M. Fraser^91^, Jackie F. Price^91^, Krista Fischer^10^, Kaarel Krjutaikov^10^, Andres Metspalu^10^, Evelin Mihailov^10,11^, Claudia Langenberg^9,157^, Jian'an Luan^9^, Ken K. Ong^9,158^, Peter S. Chines^59^, Sirkka M. Keinanen-Kiukaanniemi^159,160^, Timo E. Saaristo^161,162^, Sarah Edkins^1^, Paul W. Franks^163,164,165^, Goran Hallmans^165^, Dmitry Shungin^163,165,166^, Andrew David Morris^167^, Colin N. A. Palmer^167^, Raimund Erbel^168^, Susanne Moebus^17^, Markus M. Nothen^169,170^, Sonali Pechlivanis^17^, Kristian Hveem^171^, Narisu Narisu^59^, Anders Hamsten^172^, Steve E. Humphries^173^, Rona J. Strawbridge^172^, Elena Tremoli^174^, Harald Grallert^175^, Barbara Thorand^176^, Thomas Illig^175,177^, Wolfgang Koenig^178^, Martina Muller-Nurasyid^74,179,180^, Annette Peters^176^, Bernhard O. Boehm^181^, Marcus E. Kleber^182,183^, Winfried Marz^183,184^, Bernhard R. Winkelmann^185^, Johanna Kuusisto^186^, Markku Laakso^186^, Dominique Arveiler^187^, Giancarlo Cesana^188^, Kari Kuulasmaa^66^, Jarmo Virtamo^66^, John W. G. Yarnell^189^, Diana Kuh^158^, Andrew Wong^158^, Lars Lind^190^, Ulf de Faire^191^, Bruna Gigante^191^, Patrik K. E. Magnusson^16^, Nancy L. Pedersen^16^, George Dedoussis^192^, Maria Dimitriou^192^, Genovefa Kolovou^193^, Stavroula Kanoni^1^, Kathleen Stirrups^1^, Lori L. Bonnycastle^59^, Inger Njølstad^194^, Tom Wilsgaard1^94^, Andrea Ganna^16^, Emil Rehnberg^16^, Aroon Hingorani^157^, Mika Kivimaki^157^, Meena Kumari^157^, Themistocles L. Assimes^195^, Ines Barroso^1,196^, Michael Boehnke^7^, Ingrid B. Borecki1^4^, Panos Deloukas^1^, Caroline S. Fox^197^, Timothy Frayling^21^, Leif C. Groop^198^, Talin Haritunians^199^, David Hunter^13,22,200^, Erik Ingelsson^16^, Robert Kaplan^201^, Karen L. Mohlke^15^, Jeffrey R. O'Connell^39^, David Schlessinger^202^, David P. Strachan^203^, Kari Stefansson^24,47^, Cornelia M. van Duijn^49,52,204^, Gonc çalo R. Abecasis^7^, Mark I. McCarthy^2,27,205^, Joel N. Hirschhorn^18,19,20^, Lu Qi^13,200^, Ruth J. F. Loos^9,206^, Cecilia M. Lindgren^2^, Kari E. North^8^, Iris M. Heid^3,74^

^1^Wellcome Trust Sanger Institute, Hinxton, Cambridge, UK, ^2^Wellcome Trust Centre for Human Genetics, University of Oxford, Oxford, UK, ^3^Department of Genetic Epidemiology, Institute of Epidemiology and Preventive Medicine, University of Regensburg, Regensburg, Germany, ^4^Department of Medical Genetics, University of Lausanne, Lausanne, Switzerland, ^5^Swiss Institute of Bioinformatics, Lausanne, Switzerland, ^6^Division of Cancer Epidemiology and Genetics, National Cancer Institute, National Institutes of Health, Department of Health and Human Services, Bethesda, MD, USA, ^7^Department of Biostatistics, Center for Statistical Genetics, University of Michigan, Ann Arbor, MI, USA, ^8^Department of Epidemiology, School of Public Health, University of North Carolina at Chapel Hill, Chapel Hill, NC, USA, ^9^MRC Epidemiology Unit, Institute of Metabolic Science, Addenbrooke's Hospital, Cambridge, UK, ^10^Estonian Genome Center, University of Tartu, Tartu, Estonia, ^11^Institute of Molecular and Cell Biology, University of Tartu, Tartu, Estonia, ^12^Department of Epidemiology, Tulane School of Public Health and Tropical Medicine, New Orleans, LA, USA, ^13^Department of Nutrition, Harvard School of Public Health, Boston, MA, USA, ^14^Department of Genetics, Washington University School of Medicine, St. Louis, MI, USA, ^15^Department of Genetics, University of North Carolina, Chapel Hill, NC, USA, ^16^Department of Medical Epidemiology and Biostatistics, Karolinska Institutet, Stockholm, Sweden, ^17^Institute for Medical Informatics, Biometry and Epidemiology (IMIBE), University Hospital of Essen, University of Duisburg-Essen, Essen, Germany, ^18^Divisions of Genetics and Endocrinology and Program in Genomics, Children's Hospital, Boston, MA, USA, ^19^Metabolism Initiative and Program in Medical and Population Genetics, Broad Institute, Cambridge, MA, USA, ^20^Department of Genetics, Harvard Medical School, Boston, MA, USA, ^21^Genetics of Complex Traits, Peninsula College of Medicine and Dentistry, University of Exeter, Exeter, UK, ^22^Department of Epidemiology, Harvard School of Public Health, Boston, MA, USA, ^23^Department of Biostatistics, Harvard School of Public Health, Boston, MA, USA, ^24^deCODE Genetics, Reykjavik, Iceland, ^25^Department of Genetic Medicine and Development, University of Geneva Medical School, Geneva, Switzerland, ^26^Biomedical Sciences Research Center Al. Fleming, Vari, Greece, ^27^Oxford Centre for Diabetes, Endocrinology and Metabolism, University of Oxford, Oxford, UK, ^28^Department of Statistics, University of Oxford, Oxford, UK, ^29^MRC Harwell, Harwell, UK, ^30^University of Texas Southwestern Medical Center, Dallas, TX, USA, ^31^Regensburg University Medical Center, Innere Medizin I, Regensburg, Germany, ^32^CNRS UMR8199-IBL-Institut Pasteur de Lille, Lille, France, ^33^Department of Genomics of Common Disease, School of Public Health, Imperial College London, London, UK, ^34^Department of Biological Psychology, VU University Amsterdam, Amsterdam, The Netherlands, ^35^Hudson Alpha Institute for Biotechnology, Huntsville, AL, USA, ^36^Laboratory of Epidemiology, Demography, Biometry, National Institute on Aging, National Institutes of Health, Bethesda, MD, USA, ^37^Icelandic Heart Association, Kopavogur, Iceland, ^38^University of Iceland, Reykjavik, Iceland, ^39^Department of Medicine, University of Maryland School of Medicine, Baltimore, MD, USA, ^40^Geriatrics Research and Education Clinical Center, Baltimore Veterans Administration Medical Center, Baltimore, MD, USA, ^41^School of Social and Community Medicine, University of Bristol, Bristol, UK, ^42^Clinical Pharmacology and Barts and The London Genome Centre, William Harvey Research Institute, Barts and The London School of Medicine and Dentistry, Queen Mary University of London, London, UK, ^43^Department of OB/GYN and Medical Genetics Institute, Cedars-Sinai Medical Center, Los Angeles, CA, USA, ^44^Department of Medicine, David Geffen School of Medicine at University of California, Los Angeles, CA, USA, ^45^Cardiovascular Health Research Unit, University of Washington, Seattle, WA, USA, ^46^Service of Medical Genetics, Centre Hospitalier Universitaire Vaudois (CHUV) University Hospital, Lausanne, Switzerland, ^47^Faculty of Medicine, University of Iceland, Reykjav ik, Iceland, ^48^Department of Public Health and Primary Care, Institute of Public Health, University of Cambridge, Cambridge, UK, ^49^Department of Epidemiology, Erasmus MC, Rotterdam, The Netherlands, ^50^Department of Clinical Genetics, Erasmus MC, Rotterdam, The Netherlands, ^51^Centre for Medical Systems Biology & Netherlands Consortium on Healthy Aging, Leiden, The Netherlands, ^52^Netherlands Genomics Initiative (NGI)-sponsored Netherlands Consortium for Healthy Aging (NCHA), Leiden, The Netherlands, ^53^Department of Biostatistics, Boston University School of Public Health, Boston, MA, USA, ^54^Department of Neurology, Boston University School of Medicine, Boston, MA, USA, ^55^National Institute for Health and Welfare, Unit for Child and Adolescent Psychiatry, Helsinki, Finland, ^56^Finnish Twin Cohort Study, Department of Public Health, University of Helsinki, Helsinki, Finland, ^57^Institute for Molecular Medicine Finland (FIMM), University of Helsinki, Helsinki, Finland, ^58^National Institute for Health and Welfare, Department of Chronic Disease Prevention, Unit of Public Health Genomics, Helsinki, Finland, ^59^Genome Technology Branch, National Human Genome Research Institute, NIH, Bethesda, MD, USA, ^60^South Karelia Central Hospital, Lappeenranta, Finland, ^61^Red RECAVA Grupo RD06/0014/0015, Hospital Universitario, La Paz, Madrid, Spain, ^62^Centre for Vascular Prevention, Danube-University Krems, Krems, Austria, ^63^National Institute for Health and Welfare, Diabetes Prevention Unit, Helsinki, Finland, ^64^South Ostrobothnia Central Hospital, Seinajoki, Finland, ^65^National Institute for Health and Welfare, Department of Chronic Disease Prevention, Population Studies Unit, Turku, Finland, ^66^National Institute for Health and Welfare, Department of Chronic Disease Prevention, Chronic Disease Epidemiology and Prevention Unit, Helsinki, Finland, ^67^Nordic Center of Cardiovascular Research (NCCR), Lu¨beck, Germany, ^68^Universita¨t zu Lu¨beck, Medizinische Klinik II, Lu¨beck, Germany, ^69^Institut fu¨r Medizinische Biometrie und Statistik, Universita¨t zu Lu¨beck, Universita¨tsklinikum Schleswig-Holstein, Campus Lu¨beck, Lu¨beck, Germany, ^70^Deutsches Herzzentrum Mu¨nchen and DZHK (German Center for Cardiovascular Research), partner site Munich Heart Alliance, Munich, Germany, ^71^Division of Genetic Epidemiology, Department of Medical Genetics, Molecular and Clinical Pharmacology, Innsbruck Medical University, Innsbruck, Austria, ^72^Institute of Epidemiology I, Helmholtz Zentrum Mu¨nchen – German Research Center for Environmental Health, Neuherberg, Germany, ^73^Institute of Medical Informatics, Biometry and Epidemiology, Chair of Epidemiology, Ludwig-Maximilians-Universita¨t, and Klinikum Grosshadern, Munich, Germany, ^74^Institute of Genetic Epidemiology, Helmholtz Zentrum Mu¨nchen – German Research Center for Environmental Health, Neuherberg, Germany, ^75^Center for Biomedicine, European Academy Bozen/Bolzano (EURAC), Bolzano/Bozen, Italy, Affiliated Institute of the University of Lu¨beck, Lu¨beck, Germany, ^76^Department of Immunology, Genetics and Pathology, Uppsala University, Uppsala, Sweden, ^77^Uppsala Clinical Research Center, Uppsala University Hospital, Uppsala, Sweden, ^78^Department of Neurology, General Central Hospital, Bolzano, Italy, ^79^Department of Neurology, University of Lu¨beck, Lu¨beck, Germany, ^80^Cardiovascular Research Center and Cardiology Division, Massachusetts General Hospital, Boston, MA, USA, ^81^Center for Human Genetic Research, Massachusetts General Hospital, Boston, MA, USA, ^82^Program in Medical and Population Genetics, Broad Institute of Harvard and Massachusetts Institute of Technology, Cambridge, MA, USA, ^83^Center for Computational Medicine and Bioinformatics, University of Michigan, Ann Arbor, MI, USA, ^84^Department of Internal Medicine, Division of Gastroenterology, University of Michigan, Ann Arbor, MI, USA, ^85^Department of Psychiatry, University Medical Centre Groningen, Groningen, The Netherlands, ^86^Department of Clinical Sciences/Obstetrics and Gynecology, University of Oulu, Oulu, Finland, ^87^Department of Epidemiology and Biostatistics, School of Public Health, Faculty of Medicine, Imperial College London, London, UK, ^88^Institute of Health Sciences, University of Oulu, Oulu, Finland, ^89^Biocenter Oulu, University of Oulu, Oulu, Finland, ^90^National Institute for Health and Welfare, Oulu, Finland, ^91^Centre for Population Health Sciences, University of Edinburgh, Edinburgh, UK, ^92^Cardiovascular Medicine, University of Oxford, Wellcome Trust Centre for Human Genetics, Oxford, UK, ^93^Department of Internal Medicine, Erasmus MC, Rotterdam, The Netherlands, ^94^Department of Internal Medicine, VU University Medical Centre, Amsterdam, The Netherlands, ^95^Department of Epidemiology, Biostatistics and HTA, Radboud University Nijmegen Medical Centre, Nijmegen, The Netherlands, ^96^Department of Urology, Radboud University Nijmegen Medical Centre, Nijmegen, The Netherlands, ^97^Comprehensive Cancer Center East, Nijmegen, The Netherlands, ^98^Istituto di Neurogenetica e Neurofarmacologia del CNR, Monserrato, Cagliari, Italy, ^99^Department of Oncology, University of Cambridge, Cambridge, UK, ^100^Interfaculty Institute for Genetics and Functional Genomics, Ernst-Moritz-Arndt-University Greifswald, Greifswald, Germany, ^101^Institute for Community Medicine, Ernst-Moritz-Arndt-University Greifswald, Greifswald, Germany, ^102^Interdisciplinary Centre for Clinical Research, University of Leipzig, Leipzig, Germany, ^103^University of Leipzig, IFB Adiposity Diseases, Leipzig, Germany, ^104^Department of Medicine, University of Leipzig, Leipzig, Germany, ^105^Department of Twin Research and Genetic Epidemiology, King's College London, London, UK, ^106^MRC Human Genetics Unit, Institute for Genetics and Molecular Medicine, Western General Hospital, Edinburgh, UK, ^107^Division of Cardiovascular and Neuronal Remodelling, Multidisciplinary Cardiovascular Research Centre, Leeds Institute of Genetics, Health and Therapeutics, University of Leeds, Leeds, UK, ^108^Department of Cardiovascular Sciences, University of Leicester, Glenfield Hospital, Leicester, UK, ^109^Leicester NIHR Biomedical Research Unit in Cardiovascular Disease, Glenfield Hospital, Leicester, UK, ^110^Department of Haematology, University of Cambridge, Cambridge, UK, ^111^NHS Blood and Transplant, Cambridge Centre, Cambridge, UK, ^112^School of Medicine and Pharmacology, The University of Western Australia, Nedlands, WA, Australia, ^113^Busselton Population Medical Research Foundation, Inc., Sir Charles Gairdner Hospital, Nedlands, Western Australia, Australia, ^114^Genetic Epidemiology and Biostatistics Platform, Ontario Institute for Cancer Research, Toronto, Canada, ^115^Prosserman Centre for Health Research, Samuel Lunenfeld Research Institute, Toronto, Canada, ^116^Transplantation Laboratory, Haartman Institute, University of Helsinki, Helsinki, Finland, ^117^Division of Cardiology, Cardiovascular Laboratory, Helsinki University Central Hospital, Helsinki, Finland, ^118^Montreal Heart Institute, Montreal, QC, Canada, ^119^Institute of Clinical Medicine, Department of Internal Medicine, University of Oulu, Oulu, Finland, ^120^Department of Internal Medicine, Institute of Medicine, Sahlgrenska Academy, University of Gothenburg, Gothenburg, Sweden, ^121^Department of General Practice and Primary Health Care, University of Helsinki, Helsinki, Finland, ^122^National Institute for Health and Welfare, Helsinki, Finland, ^123^Helsinki University Central Hospital, Unit of General Practice, Helsinki, Finland, ^124^University of Milan, Department of Medicine, Surgery and Dentistry, Milano, Italy, ^125^Unit of Genetic Epidemiology and Bioinformatics, Department of Epidemiology, University Medical Center Groningen, University of Groningen, Groningen, The Netherlands, ^126^LifeLines Cohort Study, University Medical Center Groningen, University of Groningen, Groningen, The Netherlands, ^127^Department of Endocrinology, University Medical Center Groningen, University of Groningen, Groningen, The Netherlands, ^128^University of Chicago, Chicago, IL, USA, ^129^Northshore University Healthsystem, Evanston, IL, USA, ^130^Core Genotyping Facility, SAIC-Frederick, Inc., NCI-Frederick, Frederick, MD, USA, ^131^Department of Internal Medicine, University Medical Center Groningen, University of Groningen, Groningen, The Netherlands, ^132^Department of Cardiology, University Medical Center Groningen, University of Groningen, Groningen, The Netherlands, ^133^Department of Genetics, University Medical Center Groningen, University of Groningen, Groningen, The Netherlands, ^134^Genetic Epidemiology Laboratory, Queensland Institute of Medical Research, QLD, Australia, ^135^Molecular Epidemiology Laboratory, Queensland Institute of Medical Research, QLD, Australia, ^136^Queensland Statistical Genetics Laboratory, Queensland Institute of Medical Research, QLD, Australia, ^137^Division of Preventive Medicine, Brigham and Women's Hospital, Boston, MA, USA, ^138^Harvard Medical School, Boston, MA, USA, ^139^Department of Clinical Chemistry, University of Tampere and Tampere University Hospital, Tampere, Finland, ^140^Research Centre of Applied and Preventive Cardiovascular Medicine, University of Turku, Turku, Finland, ^141^The Department of Clinical Physiology, Turku University Hospital, Turku, Finland, ^142^Division of Research, Kaiser Permanente Northern California, Oakland, CA, USA, ^143^Department of Vascular Medicine, Academic Medical Center, Amsterdam, The Netherlands, ^144^Centre For Paediatric Epidemiology and Biostatistics/MRC Centre of Epidemiology for Child Health, University College of London Institute of Child Health, London, UK, ^145^Telethon Institute for Child Health Research, West Perth, WA, Australia, ^146^Centre for Child Health Research, The University of Western Australia, Perth, Australia, ^147^PathWest Laboratory of Western Australia, Department of Molecular Genetics, QEII Medical Centre, Nedlands, WA, Australia, ^148^School of Pathology and Laboratory Medicine, University of Western Australia, Nedlands, WA, Australia, ^149^School of Population Health, The University of Western Australia, Nedlands, WA, Australia, ^150^Medizinische Klinik II, Universita¨ t zu Lu¨beck, Lu¨beck, Germany, ^151^Department of Medicine III, University of Dresden, Medical Faculty Carl Gustav Carus, Dresden, Germany, ^152^Department of Public Health and Clinical Nutrition, University of Eastern Finland, Kuopio, Finland, ^153^Research Unit, Kuopio University Hospital, Kuopio, Finland, ^154^Institute of Biomedicine/Physiology, University of Eastern Finland, Kuopio Campus, Kuopio, Finland, ^155^Kuopio Research Institute of Exercise Medicine, Kuopio, Finland, ^156^Department of Clinical Physiology and Nuclear Medicine, Kuopio University Hospital, Kuopio, Finland, ^157^Department of Epidemiology and Public Health, University College London, London, UK, ^158^MRC Unit for Lifelong Health & Ageing, London, UK, ^159^Faculty of Medicine, Institute of Health Sciences, University of Oulu, Oulu, Finland, ^160^Unit of General Practice, Oulu University Hospital, Oulu, Finland, ^161^Finnish Diabetes Association, Tampere, Finland, ^162^Pirkanmaa Hospital District, Tampere, Finland, ^163^Department of Clinical Sciences, Genetic and Molecular Epidemiology Unit, Skåne University Hospital Malmo¨, Lund University, Malmo¨, Sweden, ^164^Department of Nutrition, Harvard School of Public Health, Boston, MA, USA, ^165^Department of Public Health & Clinical Medicine, Umeå University, Umeå, Sweden, ^166^Department of Odontology, Umeå University, Umea, Sweden, ^167^Medical Research Institute, University of Dundee, Ninewells Hospital and Medical School, Dundee, UK, ^168^Clinic of Cardiology, West German Heart Centre, University Hospital of Essen, University Duisburg-Essen, Essen, Germany, ^169^Institute of Human Genetics, University of Bonn, Bonn, Germany, ^170^Department of Genomics, Life & Brain Center, University of Bonn, Bonn, Germany, ^171^HUNT Research Centre, Department of Public Health and General Practice, Norwegian University of Science and Technology, Levanger, Norway, ^172^Atherosclerosis Research Unit, Department of Medicine, Solna, Karolinska Institutet, Karolinska University Hospital, Stockholm, Sweden, ^173^Cardiovascular Genetics, British Heart Foundation Laboratories, Rayne Building, University College London, London, UK, ^174^Department of Pharmacological Sciences, University of Milan, Monzino Cardiology Center, IRCCS, Milan, Italy, ^175^Unit for Molecular Epidemiology, Helmholtz Zentrum Munchen – German Research Center for Environmental Health, Neuherberg, Germany, ^176^Institute of Epidemiology II, Helmholtz Zentrum Munchen – German Research Center for Environmental Health, Neuherberg, Germany, ^177^Hannover Unified Biobank, Hannover Medical School, Hannover, Germany, ^178^Department of Internal Medicine II – Cardiology, University of Ulm Medical Center, Ulm, Germany, ^179^Department of Medicine I, University Hospital Grosshadern, Ludwig-Maximilians-Universitat, Munich, Germany, ^180^Institute of Medical Informatics, Biometry and Epidemiology, Chair of Genetic Epidemiology, Ludwig-Maximilians-Universitat, Munich, Germany, ^181^Division of Endocrinology and Diabetes, Department of Medicine, University Hospital, Ulm, Germany, ^182^LURIC Study nonprofit LLC, Freiburg, Germany, ^183^Mannheim Institute of Public Health, Social and Preventive Medicine, Medical Faculty of Mannheim, University of Heidelberg, Mannheim, Germany, ^184^Synlab Academy, Mannheim, Germany, ^185^Cardiology Group, Frankfurt-Sachsenhausen, Germany, ^186^Department of Medicine, University of Kuopio and Kuopio University Hospital, Kuopio, Finland, ^187^Department of Epidemiology and Public Health, Faculty of Medicine, Strasbourg, France, ^188^Department of Clinical Medicine, University of Milano-Bicocca, Monza, Italy, ^189^Centre for Public Health, Queen's University, Belfast, UK, ^190^Department of Medical Sciences, Uppsala University, Akademiska Sjukhuset, Uppsala, Sweden, ^191^Division of Cardiovascular Epidemiology, Institute of Environmental Medicine, Karolinska Institutet, Stockholm, Sweden, ^192^Department of Dietetics-Nutrition, Harokopio University, Athens, Greece, ^193^First Cardiology Department, Onassis Cardiac Surgery Center, Athens, Greece, ^194^Department of Community Medicine, Faculty of Health Sciences, University of Tromsø, Tromsø, Norway, ^195^Department of Medicine, Stanford University School of Medicine, Stanford, CA, USA, ^196^University of Cambridge Metabolic Research Labs, Institute of Metabolic Science Addenbrooke's Hospital, Cambridge, UK, ^197^Division of Intramural Research, National Heart, Lung and Blood Institute, Framingham Heart Study, Framingham, MA, USA, ^198^Lund University Diabetes Centre, Department of Clinical Sciences, Lund University, Malmo¨, Sweden, ^199^Medical Genetics Institute, Cedars-Sinai Medical Center, Los Angeles, CA, USA, ^200^Channing Laboratory, Department of Medicine, Brigham and Women's Hospital and Harvard Medical School, Boston, MA, USA, ^201^Department of Epidemiology and Population Health, Albert Einstein College of Medicine, Bronx, NY, USA, ^202^Laboratory of Genetics, National Institute on Aging, Baltimore, MD, USA, ^203^Division of Community Health Sciences, St George's, University of London, London, UK, ^204^Center of Medical Systems Biology, Leiden University Medical Center, Leiden, The Netherlands, ^205^Oxford National Institute for Health Research Biomedical Research Centre, Churchill Hospital, Oxford, UK, ^206^Genetics of Obesity and Related Metabolic Traits Program, The Charles Bronfman Institute of Personalized Medicine, Child Health and Development Institute, Mount Sinai School of Medicine, NY, USA.
