# Supplementary material for: Genome-wide enrichment analysis between endometriosis and obesity-related traits reveals novel susceptibility loci
Source: Hum Mol Genet. 2014 Oct 8;24(4):1185–99. doi: 10.1093/hmg/ddu516 (PMC4576730; doi:10.1093/hmg/ddu516)
Supplement: Supplementary Data [file supp_24_4_1185__index.html]

Genome-wide enrichment analysis between endometriosis and obesity-related traits reveals novel susceptibility loci — Genome-wide enrichment analysis between endometriosis and obesity-related traits reveals novel susceptibility loci — Supplementary Data 

# Genome-wide enrichment analysis between endometriosis and obesity-related traits reveals novel susceptibility loci

## Supplementary Data

Supplementary Data

**Files in this Data Supplement:**

- Supplementary Data - Docx file
