# Supplementary material for: Genome-wide enrichment analysis between endometriosis and obesity-related traits reveals novel susceptibility loci
Source: Hum Mol Genet. 2014 Oct 8;24(4):1185–99. doi: 10.1093/hmg/ddu516 (PMC4576730; doi:10.1093/hmg/ddu516)
Supplement: Supplementary Data [file supp_ddu516_ddu516supp.docx]

**SUPPLEMENTARY INFORMATION**

**Contents**

**Supplementary Figures and Tables**

**Supplementary Figure 1.** Female-limited genetic enrichment analyses between endometriosis, BMI and WHRadjBMI GWAS datasets, using independent (r^2^<0.2) SNPs. The panels show: (1) The proportion of SNPs nominally associated (P<0.05) with WHRadjBMI (Panel A) or BMI (Panel B) by significance of overall and stage B endometriosis association (P<1.0 x 10^-3^ vs. P≥1x10^-3^); (2) The proportion of SNPs nominally associated (P<0.05) with overall and stage B endometriosis by significance of WHRadjBMI (Panel C) and BMI (Panel D) association (P<1.0 x 10^-3^ vs. P≥1x10^-3^). P-values of chi^2^ tests assessing statistical difference between proportions are shown above each set of bars, and 95% confidence intervals of the proportions are given on each bar. For differences with P_chisq_ < 0.2, empirical p-values are given in brackets (see Methods).

**Supplementary Figure 2**. Directions of effect of SNPs associated with all (panel A) and stage B endometriosis (panel B) (P<0.001) and WHRadjBMI (P<0.05). Linear regression R^2^ and p-values used to test for significant directionality of effects are shown.

**Supplementary Figure 3**. Graphical illustration of the GRAIL results: a. Overall endometriosis and WHRadjBMI, 79 genes were detected marked by 72 independent SNPs, b. Stage B endometriosis and WHRadjBMI, 77 genes were detected marked by 85 independent SNPs.

**Supplementary Figure 4.** Q-Q plots of observed vs. expected GWAS results for a. Overall endometriosis, b. Stage B endometriosis.

**Supplementary Table 1.** Association results of published IEC genome-wide significant endometriosis loci([1](#_ENREF_1)) in the GIANT BMI GWAS, and of BMI loci([2](#_ENREF_2)) in endometriosis GWAS (look-up results are shown in bold).

**Supplementary Table 2.** Contingency tables that are the basis of the chi^2^ test for genetic enrichment (Figure 1), showing the numbers (proportions) of independent SNPs (r2 >0.2) associated with WHRadjBMI at P<0.05 vs. P≥0.05 by association P-value (P<1x10^-3^ vs. P≥ 1 x 10^-3^) for overall (**a**) and stage B (**b**) endometriosis.

**Supplementary Table 3**. Sensitivity analysis testing the effect of different significance thresholds for association detection in discovery (overall endometriosis) and look-up (WHRadjBMI) data-sets.

**Supplementary Table 4.** Sensitivity analysis testing the effect of different significance thresholds for association detection in discovery (overall endometriosis) and look-up (BMI) data-sets.

**Supplementary Table 5.** Polygenic prediction results of overall WHRadjBMI derived profile scores on overall endometriosis and stage B endometriosis.

**Supplementary Table 6.** Polygenic prediction results of female-limited WHRadjBMI derived profile scores on overall endometriosis and stage B endometriosis.

**Supplementary Table 7.** Polygenic prediction results of overall BMI derived profile scores on overall endometriosis and stage B endometriosis.

**Supplementary Table 8.** Polygenic prediction results of female-limited BMI derived profile scores on overall endometriosis and stage B endometriosis.

**Supplementary Table 9.** Directionality of effects of the shared SNPs (primary dataset p<1x10^-3^ and look-up dataset p<0.05) between endometriosis (all and stage B only) and BMI/WHRadjBMI GWAS results.

**Supplementary Table 10.** Directionality of effects of the shared SNPs (primary dataset p<1x10^-3^ and look-up dataset p<0.05) between endometriosis (all and stage B only) and female-limited BMI/WHRadjBMI GWAS results.

**Supplementary Table 11.** Annotation of genetic enrichment analysis results between all endometriosis and WHRadjBMI (n=52).

**Supplementary Table 12.** Annotation of genetic enrichment analysis results between stage B endometriosis and WHRadjBMI (n=57).

**Supplementary Table 13.** Results of the Mammalian Gene Expression Uterus database (MGEx-Udb)([4](#_ENREF_3)) look up for the 16 genes in human endometrium tissue from individuals with endometriosis and without.

**Supplementary Table 14.** Results of the *cis*-eQTL analysis in the MUTHER data for abdominal fat expression of the 16 genes([5](#_ENREF_4)).

**Supplementary Table 15.** Results of the differential *cis*-eQTL analysis between abdominal and gluteal fat in the MolOBB data on the 16 genes.

**Supplementary Table 16.** Results of over/under-representation of (a) 88 genes associated with all endometriosis (P<1.0x10^-3^) and WHRadjBMI (P<0.05), and (b) 103 genes associated with stage B endometriosis (P<1.0x10^-3^) and WHRadjBMI (P<0.05), in 241 biological processes in PANTHER.

**Supplementary Table 17.** Results of over/under-representation of (a) 88 genes associated with all endometriosis (P<1.0x10^-3^) and WHRadjBMI (P<0.05), and (b) 103 genes associated with stage B endometriosis (P<1.0x10^-3^) and WHRadjBMI (P<0.05), in 176 curated biological pathways in PANTHER.

**Supplementary Table 18.** Sensitivity analysis on over/under-representation of genes associated with all endometriosis and WHRadjBMI with using two additional p-value thresholds: (1) P<1x10^-4^ and (2) P<1x10^-2^ in comparison to our original enrichment threshold of p<1x10^-3^, in 176 curated biological pathways in PANTHER.

**Supplementary Table 19.** Overall endometriosis and WHRadjBMI GRAIL results (P<0.5).

**Supplementary Table 20.** Stage B endometriosis and WHRadjBMI GRAIL results (P<0.5).

**Supplementary Figure 1.** Female-limited genetic enrichment analyses between endometriosis, BMI and WHRadjBMI GWAS datasets, using independent (r^2^<0.2) SNPs. The panels show: (1) The proportion of SNPs nominally associated (P<0.05) with WHRadjBMI (Panel A) or BMI (Panel B) by significance of overall and stage B endometriosis association (P<1.0 x 10^-3^ vs. P≥1x10^-3^); (2) The proportion of SNPs nominally associated (P<0.05) with overall and stage B endometriosis by significance of WHRadjBMI (Panel C) and BMI (Panel D) association (P<1.0 x 10^-3^ vs. P≥1x10^-3^). P-values of chi^2^ tests assessing statistical difference between proportions are shown above each set of bars, and 95% confidence intervals of the proportions are given on each bar. For differences with P_chisq_ < 0.2, empirical p-values are given in brackets (see Methods).


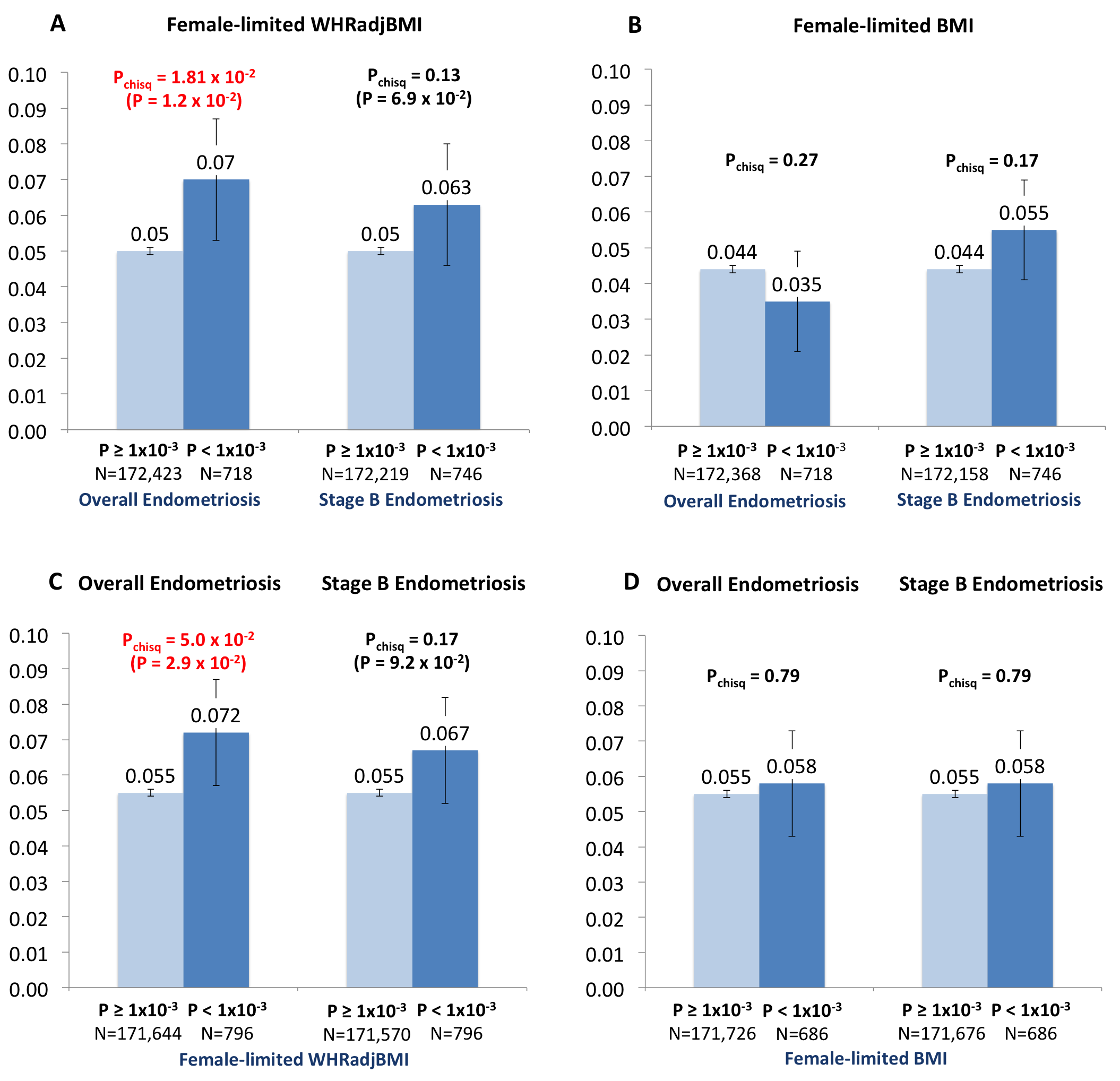


**Supplementary Figure 2**. Directions of effect of SNPs associated with all (panel A) and stage B endometriosis (panel B) (P<0.001) and WHRadjBMI (P<0.05). Linear regression R^2^ and p-values used to test for significant directionality of effects are shown.

**
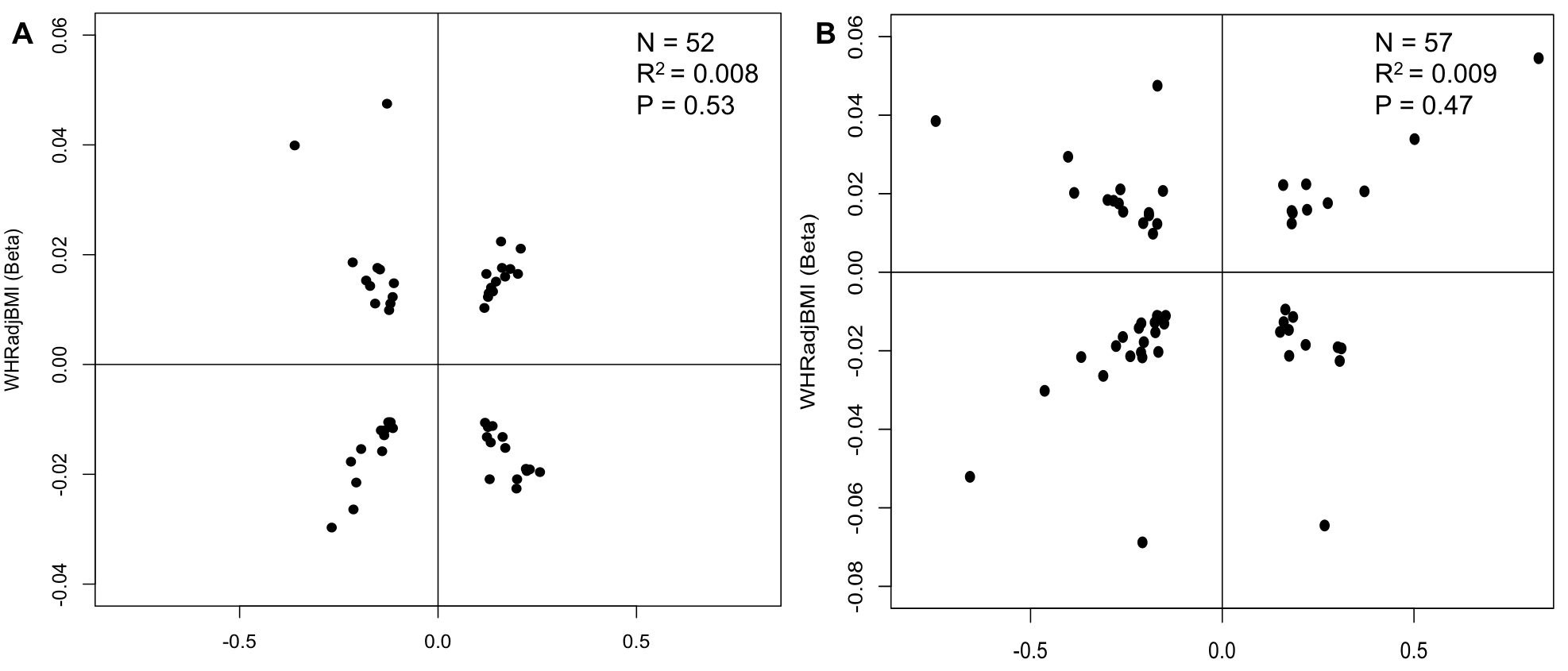
**

Overall Endometriosis (Beta)

Stage B Endometriosis (Beta)

**Supplementary Figure 3**. Graphical illustration of the GRAIL results: a. Overall endometriosis and WHRadjBMI, 79 genes were detected marked by 72 independent SNPs, b. Stage B endometriosis and WHRadjBMI, 77 genes were detected marked by 85 independent SNPs.

a.
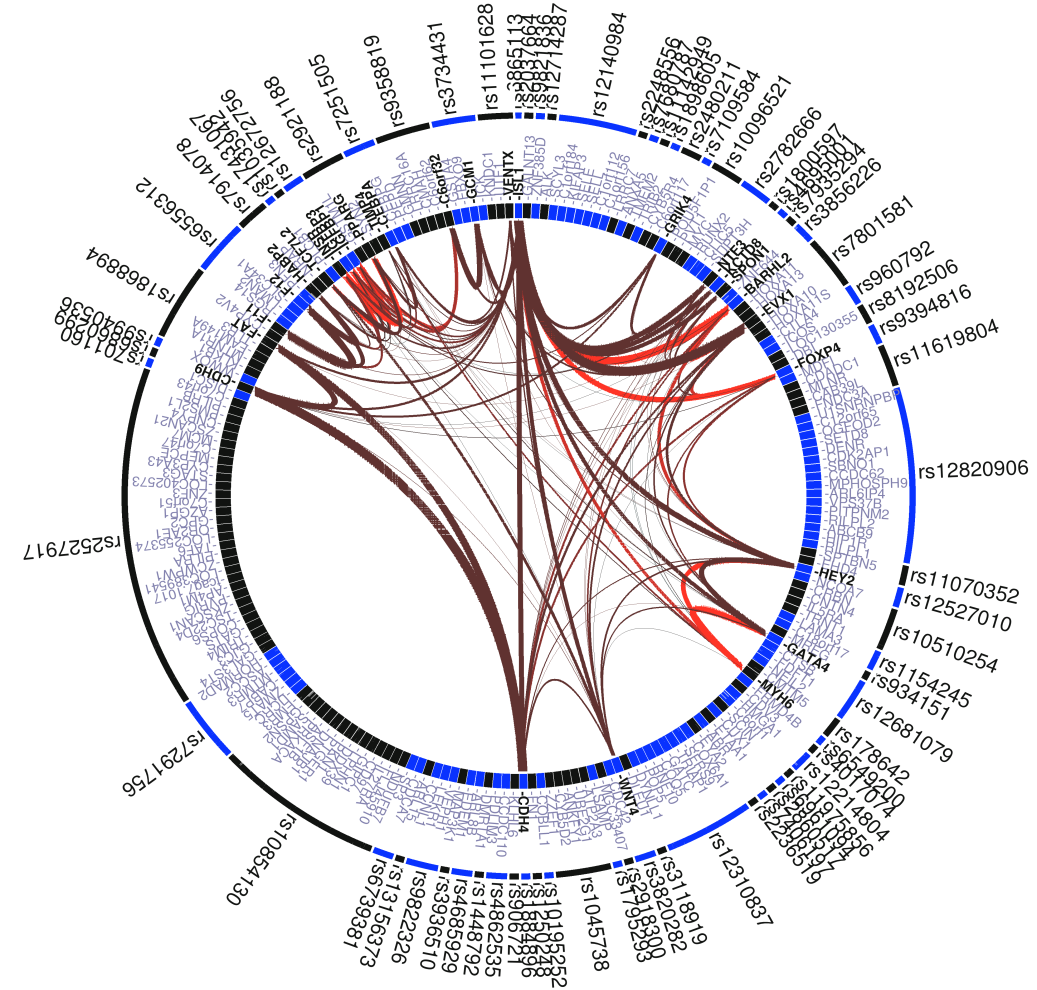


b.
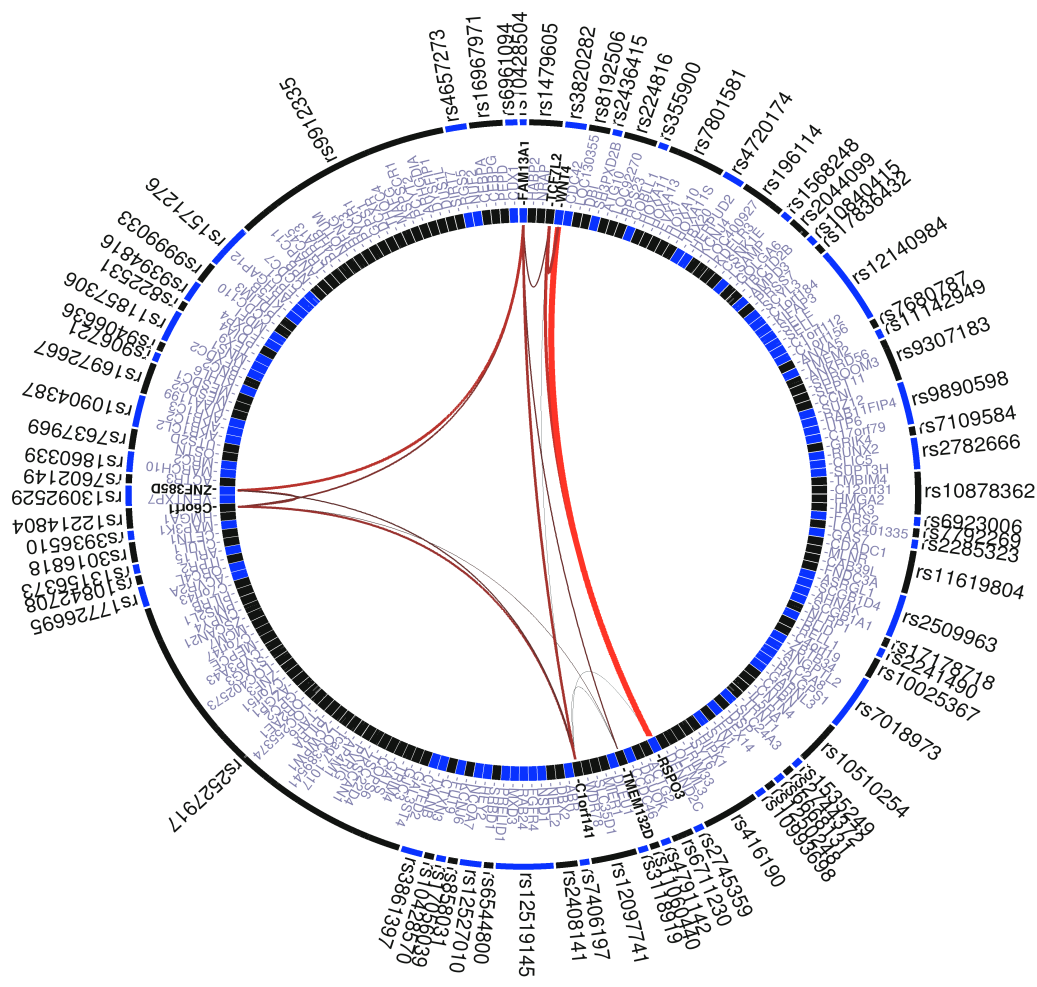


**Supplementary Figure 4.** Q-Q plots of observed vs. expected GWAS results for, a. Overall endometriosis, b. Stage B endometriosis.

**a.** Overall Endometriosis **b.** Stage B Endometriosis


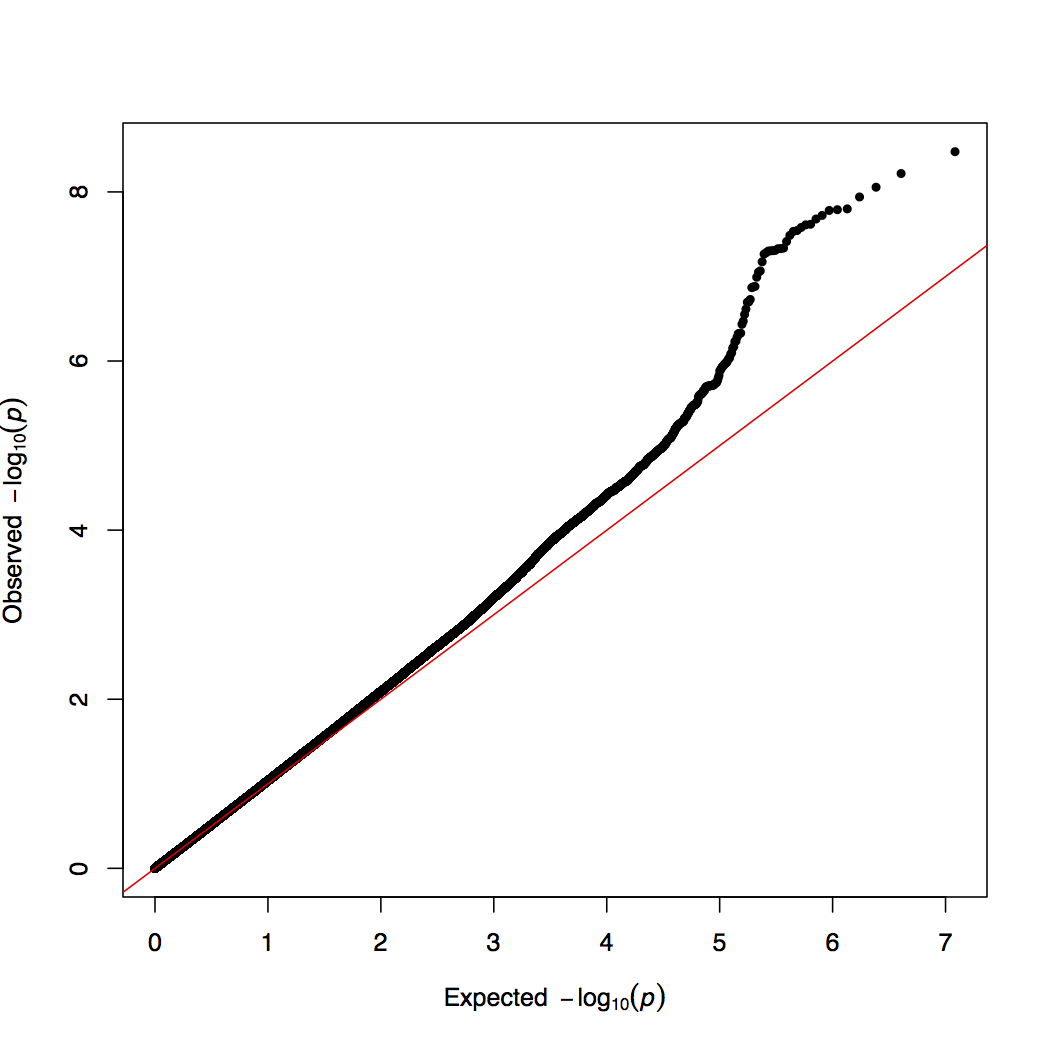

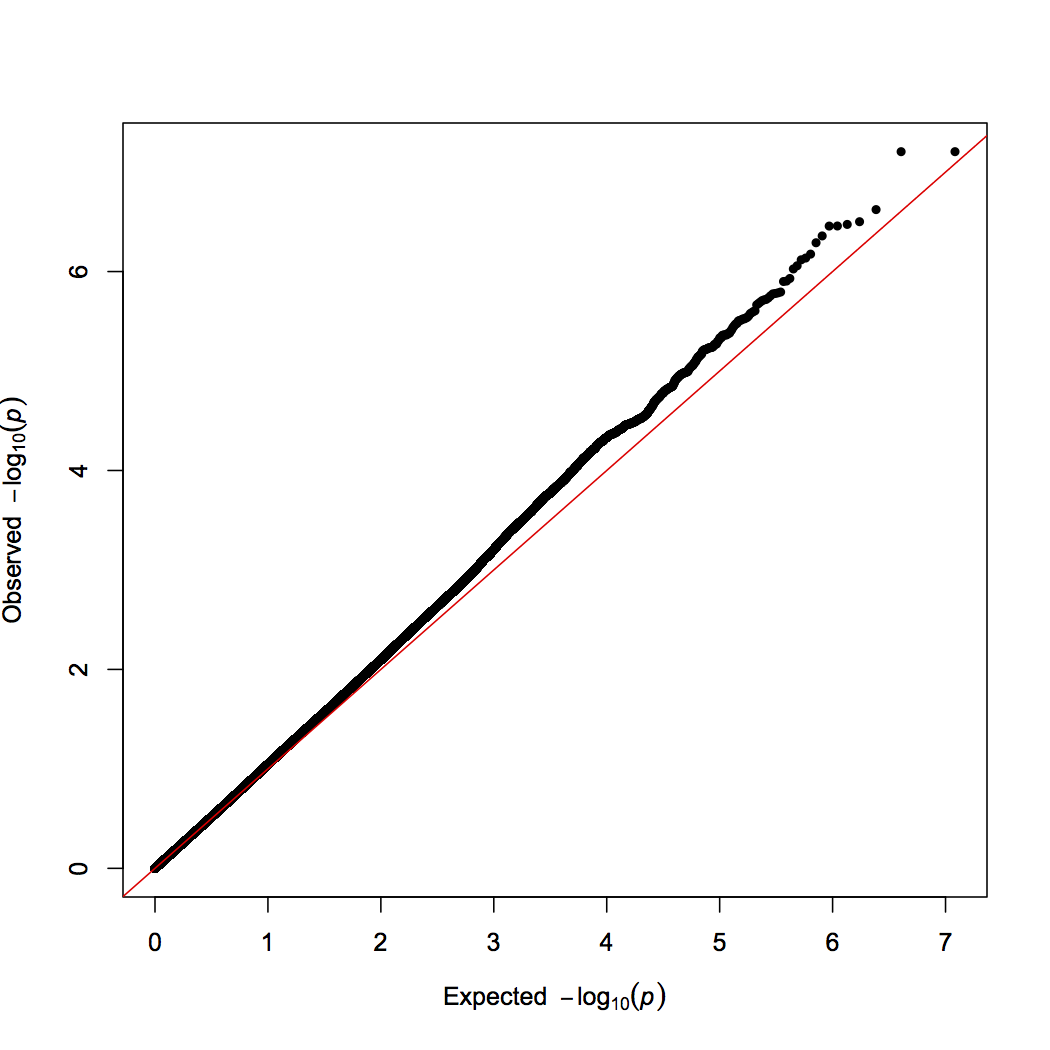


**Supplementary Table 1.** Association results of published IEC genome-wide significant endometriosis loci([1](#_ENREF_1)) in the GIANT BMI GWAS, and of BMI loci([2](#_ENREF_2)) in endometriosis GWAS (look-up results are shown in bold).

| **GWAS** | **SNP**  **(proxy; r^2^)** | **Chr** | **Location**  **(B36)** | **RAF**  **(allele)** | **Status** | **Endometriosis**  **All cases** | | **Endometriosis**  **Stage B** | | **Overall BMI** | | **Female limited BMI** | | | **Nearest**  **Gene** |
| --- | --- | --- | --- | --- | --- | --- | --- | --- | --- | --- | --- | --- | --- | --- | --- |
|  |  |  |  |  |  | **P.Val**^b^ | **OR**  **(95% CI)** | **P.Val**^b^ | **OR**  **(95% CI)** | **P.Val**^c^ | **Effect**  **(SE)** | | **P.Val**^d^ | **Effect**  **(SE)** |  |
| Endometriosis | rs12700667 | 7 | 25868164 | 0.74 (A) | G | 5.1x10^-7^ | 1.21  (1.12-1.31) | 3.3x10^-8^ | 1.36  (1.23-1.50) | **0.27** | **0.006**  **(0.005)** | | **0.15** | 0.012 (0.080) | Intergenic |
| Endometriosis | rs7521902 | 1 | 22363311 | 0.75 (A) | G | 8.9x10^-5^ | 1.16  (1.08-1.25) | 7.5x10^-5^ | 1.26  (1.14-1.39) | **0.92** | **-0.006 (0.006)** | | **0.97** | -0.001  (0.008) | *WNT4* |
| BMI | rs9816226 | 3 | 187,317,193 | 0.18 (A) | I (0.98) | **0.01** | **1.12**  **(1.03-1.22)** | **0.16** | **1.08**  **(0.97-1.21)** | 9.3x10^-14^ | -0.046  (0.006) | | 2.4x10^-8^ | -0.045  (0.008) | *ETV5* |
| BMI | rs1514175 | 1 | 74,764,232 | 0.58 (A) | G | **0.02** | **0.92**  **(0.87-0.99)** | **3.9x10^-3^** | **0.88**  **(0.80-0.96)** | 1.6x10^-9^ | 0.029 (0.005) | | 1.2x10^-8^ | 0.035  (0.006) | *TNNI3K* |
| BMI | rs543874 | 1 | 176,156,103 | 0.80 (A) | I (0.99) | **0.02** | **1.10**  **(1.02-1.19)** | **0.03** | **1.12**  **(1.01-1.25)** | 2.6x10^-13^ | -0.044  (0.006) | | 7.1x10^-12^ | -0.054  (0.008) | *SEC16B* |
| BMI | rs2890652 | 2 | 142,676,401 | 0.82 (T) | I (0.96) | **0.05** | **0.91**  **(0.81-1.00)** | **0.02** | **0.86**  **(0.74-0.98)** | 1.7x10^-7^ | -0.033 (0.006) | | 8.7x10^-4^ | -0.027  (0.008) | *LRP1B* |
| BMI | rs12444979 | 16 | 19,933,600 | 0.14 (T) | I (0.97) | **0.07** | **1.10**  **(0.99-1.21)** | **0.34** | **1.06**  **(0.93-1.21)** | 4.9x10^-11^ | -0.046 (0.007) | | 2.3x10^-6^ | -0.043  (0.009) | *GPRC5B* |
| BMI | rs10968576 | 9 | 28,404,339 | 0.32 (A) | G | **0.08** | **0.94**  **(0.87-1.01)** | **0.02** | **0.90**  **(0.81-0.99)** | 2.2x10^-8^ | -0.029 (0.005) | | 1.1x10^-5^ | -0.029  (0.007) | *LRRN6C* |
| BMI | rs987237 | 6 | 50,911,009 | 0.18 (G) | G | **0.08** | **0.93**  **(0.84-1.01)** | **0.17** | **0.93**  **(0.82-1.03)** | 7.9x10^-16^ | 0.049  (0.007) | | 1.8x10^-10^ | 0.051 (0.008) | *TFAP2B* |
| BMI | rs10150332 | 14 | 79,936,964 | 0.21 (C) | I (0.99) | **0.09** | **1.01**  **(0.93-1.09)** | **0.23** | **1.03**  **(0.93-1.14)** | 3.2x10^-7^ | 0.029  (0.006) | | 4.5x10^-4^ | 0.026  (0.008) | *NRXN3* |
| BMI | rs2241423 | 15 | 65,873,892 | 0.78 (A) | G | **0.14** | **1.06**  **(0.98-1.15)** | **0.12** | **1.09**  **(0.98-1.20)** | 1.4x10^-10^ | -0.037 (0.006) | | 2.9x10^-5^ | -0.031  (0.007) | *MAP2K5* |
| BMI | rs3817334 | 11 | 47,607,569 | 0.59 (C) | I (0.99) | **0.14** | **1.05**  **(0.98-1.12)** | **0.42** | **1.04**  **(0.95-1.13)** | 5.6x10^-11^ | -0.031  (0.005) | | 4.7x10^-7^ | -0.031  (0.006) | *MTCH2* |
| BMI | rs7138803 | 12 | 50,247,468 | 0.63 (G) | G | **0.15** | **1.05**  **(0.98-1.12)** | **0.39** | **1.04**  **(0.95-1.14)** | 4.6x10^-11^ | -0.032  (0.005) | | 1.2x10^-7^ | -0.033 (0.006) | *FAIM2* |
| BMI | rs7359397 | 16 | 28,793,160 | 0.40 (T) | I (0.99) | **0.18** | **1.05**  **(0.98-1.12)** | **0.88** | **1.01**  **(0.92-1.10)** | 2.8x10^-10^ | 0.030  (0.005) | | 3.3x10^-5^ | 0.026  (0.006) | *SH2B1* |
| BMI | rs3810291 | 19 | 47,569,003 | 0.32 (G) | I (0.90) | **0.23** | **1.05**  **(0.97-1.13)** | **0.19** | **1.07**  **(0.97-1.17)** | 1.2x10^-7^ | -0.029 (0.005) | | 7.5x10^-5^ | -0.028  (0.007) | *TMEM160* |
| BMI | rs13078807 | 3 | 85,966,840 | 0.81 (G) | G | **0.25** | **0.95**  **(0.88-1.04)** | **0.37** | **0.95**  **(0.85-1.06)** | 7.7x10^-8^ | 0.032 (0.006) | | 1.2x10^-6^ | 0.037  (0.008) | *CADM2* |
| BMI | rs713586 | 2 | 25,158,008 | 0.51 (T) | I (0.98) | **0.27** | **1.04**  **(0.97-1.11)** | **0.16** | **1.07**  **(0.86-1.02)** | 2.8x10^-7^ | -0.025 (0.007) | | 3.0x10^-8^ | -0.034  (0.006) | *RBJ* |
| BMI | rs10767664 | 11 | 27,682,562 | 0.78 (A) | I (0.98) | **0.28** | **1.04**  **(0.97-1.13)** | **0.60** | **1.03**  **(0.93-1.14)** | 7.3x10^-13^ | 0.042  (0.006) | | 2.8x10^-9^ | 0.044  (0.008) | *BDNF* |
| BMI | rs3797580^a^  (rs2112347; r^2^=1.00) | 5 | 75,015,242 | 0.63 (T) | I (0.99) | **0.32** | **1.05**  **(0.96-1.14)** | **0.53** | **1.04**  **(0.92-1.17)** | 5.3x10^-8^ | 0.026 (0.005) | | 1.9x10^-5^ | 0.027  (0.006) | *FLJ35779* |
| BMI | rs4771122 | 13 | 26,918,180 | 0.24 (A) | I (0.93) | **0.45** | **1.03**  **(0.95-1.12)** | **0.32** | **1.06**  **(0.95-1.17)** | 1.3x10^-7^ | -0.030 (0.006) | | 1.6x10^-5^ | -0.032  (0.007) | *MTIF3* |
| BMI | rs4929949 | 11 | 8,604,593 | 0.48 (T) | I (0.99) | **0.48** | **1.02**  **(0.96-1.10)** | **0.87** | **1.01**  **(0.98-1.16)** | 8.3x10^-8^ | -0.026 (0.005) | | 6.9x10^-7^ | -0.031  (0.006) | *RPL27A* |
| BMI | rs2287019 | 19 | 50,894,012 | 0.81 (T) | G | **0.49** | **1.03**  **(0.95-1.12)** | **0.82** | **0.99**  **(0.88-1.10)** | 3.6x10^-7^ | -0.036 (0.007) | | 1.7x10^-5^ | -0.038  (0.009) | *QPCTL* |
| BMI | rs4836133 | 5 | 124,332,103 | 0.47 (C) | I (0.98) | **0.51** | **1.02**  **(0.96-1.09)** | **0.26** | **1.05**  **(0.96-1.15)** | 7.7x10^-7^ | -0.243 (0.005) | | 6.8x10^-5^ | -0.025  (0.006) | *ZNF608* |
| BMI | rs571312 | 18 | 55,990,749 | 0.76 (C) | G | **0.55** | **1.02**  **(0.95-1.11)** | **0.78** | **1.10**  **(0.99-1.21)** | 1.2x10^-22^ | -0.055  (0.006) | | 5.8x10^-14^ | -0.055  (0.007) | *MC4R* |
| BMI | rs13107325 | 4 | 103,407,732 | 0.91 (C) | I (0.83) | **0.59** | **1.03**  **(0.91-1.17)** | **0.19** | **1.12**  **(0.95-1.32)** | 2.4x10^-7^ | -0.056 (0.011) | | 1.6x10^-4^ | -0.054  (0.014) | *SLC39A8* |
| BMI | rs2867125 | 2 | 612,827 | 0.17 (T) | G | **0.60** | **1.02**  **(0.94-1.12)** | **0.31** | **1.07**  **(0.95-1.19)** | 6.6x10^-22^ | -0.060  (0.006) | | 1.7x10^-17^ | -0.068  (0.008) | *TMEM18* |
| BMI | rs206936 | 6 | 34,410,847 | 0.80 (A) | I (0.98) | **0.68** | **0.98**  **(0.90-1.06)** | **0.88** | **0.99**  **(0.88-1.10)** | 5.3x10^-6^ | -0.026 (0.006) | | 3.2x10^-2^ | -0.016 (0.008) | *HMGA1* |
| BMI | rs1555543 | 1 | 96,944,797 | 0.41 (A) | I (0.99) | **0.72** | **1.01**  **(0.95-1.08)** | **0.79** | **1.01**  **(0.93-1.10)** | 8.5x10^-7^ | -0.024 (0.005) | | 1.2x10^-4^ | -0.024  (0.006) | *PTBP2* |
| BMI | rs10938397 | 4 | 44,877,284 | 0.43 (G) | I (0.99) | **0.76** | **1.01**  **(0.95-1.08)** | **0.79** | **1.01**  **(0.93-1.11)** | 5.6x10^-17^ | 0.042  (0.005) | | 1.0x10^-12^ | 0.046  (0.007) | *GNPDA2* |
| BMI | rs2815752 | 1 | 72,585,028 | 0.61 (A) | I (0.98) | **0.76** | **0.99**  **(0.92-1.06)** | **0.86** | **0.99**  **(0.90-1.08)** | 1.4x10^-14^ | 0.038  (0.005) | | 1.5x10^-8^ | 0.036  (0.006) | *NEGR1* |
| BMI | rs887912 | 2 | 59,302,877 | 0.30 (T) | I (0.97) | **0.80** | **1.01**  **(0.94-1.08)** | **0.53** | **1.03**  **(0.94-1.14)** | 2.9x10^-6^ | 0.025 (0.005) | | 2.3x10^-4^ | 0.025  (0.007) | *FANCL* |
| BMI | rs29941 | 19 | 39,001,372 | 0.32 (A) | G | **0.86** | **1.01**  **(0.94-1.08)** | **0.69** | **1.02**  **(0.93-1.12)** | 3.1x10^-9^ | -0.030  (0.005) | | 4.3x10^-7^ | -0.030  (0.006) | *KCTD15* |
| BMI | rs11847697 | 14 | 29,584,863 | 0.04 (T) | I (0.92) | **0.88** | **1.01**  **(0.85-1.21)** | **0.50** | **1.08**  **(0.86-1.37)** | 4.2x10^-9^ | 0.076 (0.013) | | 2.5x10^-6^ | 0.079  (0.017) | *PRKD1* |
| BMI | rs1558902 | 16 | 52,361,075 | 0.42 (A) | I (0.97) | **0.92** | **1.00**  **(0.94-1.07)** | **0.82** | **1.01**  **(0.92-1.11)** | 5.4x10^-62^ | 0.080  (0.005) | | 8.8x10^-34^ | 0.076  (0.006) | *FTO* |

^a^ SNP was not genotyped in the endometriosis GWAS dataset; result shown is of proxy SNP.

^b^ Results are based on an updated GWAS performed using genotype data imputed up to 1000 Genomes pilot reference panel (B36, June 2010).

^c^ Results are from the GIANT BMI discovery GWAS dataset (N=123,865); 13 of the 32 BMI loci have P > 5.0 x 10^-8^, however, they reached genome-wide significance combined with replication analyses in up to a further 125,931 individuals(2).

^d^ Results a from the GIANT BMI discovery female-limited GWAS dataset (N=73,137)(3).

**Supplementary Table 2.** Contingency tables that are the basis of the chi^2^ test for genetic enrichment (Figure 1), showing the numbers (proportions) of independent SNPs (r2 >0.2) associated with WHRadjBMI at P<0.05 vs. P≥0.05 by association P-value (P<1x10^-3^ vs. P≥ 1 x 10^-3^) for overall (**a**) and stage B (**b**) endometriosis.

**a.** Overall endometriosis and WHRadjBMI

|  | **WHRadjBMI P<0.05** | **WHRadjBMI P≥0.05** |
| --- | --- | --- |
| **Overall endometriosis P<1x10^-3^** | 52 (0.073) | 665 (0.927) |
| **Overall endometriosis P≥1x10^-3^** | 8359 (0.049) | 164081 (0.951) |
| **Chi^2^ = 8.42, P = 3.7x10^-3^** | | |

**b.** Stage B endometriosis and WHRadjBMI

|  | **WHRadjBMI P<0.05** | **WHRadjBMI P≥0.05** |
| --- | --- | --- |
| **Stage B endometriosis P<1x10^-3^** | 57 (0.076) | 691 (0.924) |
| **Stage B endometriosis P≥1x10^-3^** | 8267 (0.048) | 163980 (0.952) |
| **Chi^2^ = 12.33, P = 4.5x10^-4^** | | |

**Supplementary Table 3**. Sensitivity analysis testing the effect of different significance thresholds for association detection in discovery (overall endometriosis) and look-up (WHRadjBMI) data-sets.

| **Overall endometriosis (Discovery)** | **WHRadjBMI (Look-up)** | | | | | |
| --- | --- | --- | --- | --- | --- | --- |
|  | **P<0.05** | **P≥0.05** | **P<0.01** | **P≥0.01** | **P<0.001** | **P≥0.001** |
| **P<1x10^-2^** | 278 (0.053) | 5010 (0.947) | 64 (0.01) | 5224 (0.99) | 11 (0.002) | 5277 (0.998) |
| **P≥1x10^-2^** | 8133 (0.048) | 159736 (0.952) | 1720 (0.01) | 166149 (0.99) | 218 (0.001) | 167651 (0.999) |
| **Chi^2^ = 1.80, P = 0.18** | | | **Chi^2^ = 1.56, P = 0.21** | | **Chi^2^ = 1.82, P = 0.18** | |
| **P<1x10^-3^** | **52 (0.073)** | **665 (0.927)** | 14 (0.02) | 703 (0.98) | 2 (0.003) | 715 (0.997) |
| **P≥1x10^-3^** | **8359 (0.049)** | **164081 (0.951)** | 1770 (0.01) | 170670 (0.99) | 227 (0.001) | 172213 (0.999) |
| **Chi^2^ = 8.42, P = 3.7x10^-3^** | | | **Chi^2^ = 5.13, P = 2.3x10^-2^** | | **Chi^2^ = 0.32, P = 0.57** | |
| **P<1x10^-4^** | 8 (0.075) | 99 (0.925) | 5 (0.05) | 102 (0.95) | 1 (0.001) | 106 (0.99) |
| **P≥1x10^-4^** | 8403 (0.049) | 164647 (0.951) | 1779 (0.01) | 171271 (0.99) | 228 (0.001) | 172822 (0.99) |
| **Chi^2^ = 1.07, P = 0.30** | | | **Chi^2^ = 9.96, P = 1.6x10^-3^** | | **Chi^2^ = 0.91, P = 0.34** | |
| **P<1x10^-5^** | 3 (0.15) | 17 (0.85) | 2 (0.10) | 18 (0.90) | 1 (0.05) | 19 (0.95) |
| **P≥1x10^-5^** | 8408 (0.049) | 164729 (0.951) | 1782 (0.01) | 171355 (0.99) | 228 (0.001) | 172909 (0.999) |
| **Chi^2^ = 2.53, P = 0.11** | | | **Chi^2^ = 8.20, P = 4.2x10^-3^** | | **Chi^2^ = 8.49, P = 3.6x10^-3^** | |

**Supplementary Table 4.** Sensitivity analysis testing the effect of different significance thresholds for association detection in discovery (overall endometriosis) and look-up (BMI) data-sets.

| **Overall endometriosis (Discovery)** | **BMI (Look up)** | | | | | |
| --- | --- | --- | --- | --- | --- | --- |
|  | **P<0.05** | **P≥0.05** | **P<0.01** | **P≥0.01** | **P<0.001** | **P≥0.001** |
| **P<1x10^-2^** | 244 (0.046) | 5045 (0.954) | 58 (0.01) | 5231 (0.99) | 13 (0.002) | 5276 (0.998) |
| **P≥1x10^-2^** | 7238 (0.043) | 160696 (0.957) | 1606 (0.01) | 166328 (0.99) | 271 (0.001) | 167663 (0.999) |
| **Chi^2^ = 1.07 P = 0.30** | | | **Chi^2^ = 0.92, P = 0.34** | | **Chi^2^ = 1.75, P = 0.19** | |
| **P<1x10^-3^** | **29 (0.040)** | **688 (0.96)** | 7 (0.01) | 710 (0.99) | 1 (0.001) | 716 (0.999) |
| **P≥1x10^-3^** | **7453 (0.043)** | **165053 (0.957)** | 1657 (0.01) | 170849 (0.99) | 283 (0.001) | 172223 (0.999) |
| **Chi^2^ = 0.07, P = 0.79** | | | **Chi^2^ = 0, P = 1** | | **Chi^2^ = 0, P = 1** | |
| **P<1x10^-4^** | 5 (0.047) | 102 (0.953) | 1 (0.009) | 106 (0.991) | 1 (0.009) | 106 (0.991) |
| **P≥1x10^-4^** | 7477 | 165639 | 1663 (0.01) | 171453 (0.99) | 283 (0.001) | 172833 (0.999) |
| **Chi^2^ = 0, P = 1** | | | **Chi^2^ = 0, P = 1** | | **Chi^2^ = 0.60, P = 0.44** | |
| **P<1x10^-5^** | 1 (0.05) | 19 (0.95) | 1 (0.05) | 19 (0.95) | 1 (0.05) | 19 (0.95) |
| **P≥1x10^-5^** | 7481 (0.043) | 165722 (0.957) | 1663 (0.01) | 171540 (0.99) | 283 (0.001) | 172920 (0.999) |
| **Chi^2^ = 0, P = 1** | | | **Chi^2^ = 0.50, P = 0.48** | | **Chi^2^ = 6.67, P = 9.8x10^-3^** | |

**Supplementary Table 5.** Polygenic prediction results of overall WHRadjBMI derived profile scores on overall endometriosis and stage B endometriosis.

| **Profiles** | **Overall endometriosis & overall WHR** | | | | | | **Stage B endometriosis & overall WHR** | | | | | |
| --- | --- | --- | --- | --- | --- | --- | --- | --- | --- | --- | --- | --- |
|  | **OR** | **SE** | **z** | **P.Val** | **95% CI** | **R^2^** | **OR** | **SE** | **z** | **P.Val** | **95% CI** | **R^2^** |
| **Profile 1 (**P<0.01**)** | 1.048 | 0.103 | 0.48 | 0.631 | 0.864 - 1.271 | 0.0000 | 1.051 | 0.103 | 0.51 | 0.610 | 0.867 - 1.275 | 0.0000 |
| **Profile 2 (**P<0.05**)** | 1.169 | 0.258 | 0.71 | 0.477 | 0.759 - 1.803 | 0.0000 | 1.175 | 0.259 | 0.73 | 0.466 | 0.762 - 1.810 | 0.0000 |
| **Profile 3 (**P<0.10**)** | 1.407 | 0.435 | 1.11 | 0.269 | 0.768 - 2.581 | 0.0001 | 1.412 | 0.436 | 1.12 | 0.265 | 0.770 - 2.587 | 0.0001 |
| **Profile 4 (**P<0.20**)** | 1.745 | 0.790 | 1.23 | 0.218 | 0.719 - 4.241 | 0.0001 | 1.753 | 0.793 | 1.24 | 0.214 | 0.723 - 4.254 | 0.0001 |
| **Profile 5 (**P<0.30**)** | 1.808 | 1.041 | 1.03 | 0.303 | 0.586 - 5.586 | 0.0001 | 1.821 | 1.046 | 1.04 | 0.297 | 0.591 - 5.615 | 0.0001 |
| **Profile 6 (**P<0.40**)** | 1.665 | 1.158 | 0.73 | 0.464 | 0.426 - 6.509 | 0.0000 | 1.675 | 1.163 | 0.74 | 0.458 | 0.423 - 6.533 | 0.0000 |
| **Profile 7 (**P<0.50**)** | 1.713 | 1.386 | 0.66 | 0.506 | 0.351 - 8.369 | 0.0000 | 1.706 | 1.379 | 0.66 | 0.508 | 0.350 - 8.314 | 0.0000 |
| **Profile 8 (**P<0.75**)** | 2.893 | 3.125 | 0.98 | 0.325 | 0.348 - 24.03 | 0.0001 | 2.892 | 3.125 | 0.98 | 0.325 | 0.348 - 24.03 | 0.0001 |
| **Profile 9 (**P<1.00**)** | 3.849 | 4.995 | 1.04 | 0.299 | 0.303 - 48.98 | 0.0001 | 3.894 | 5.044 | 1.05 | 0.294 | 0.308 - 49.30 | 0.0001 |

**Supplementary Table 6.** Polygenic prediction results of female-limited WHRadjBMI derived profile scores on overall endometriosis and stage B endometriosis.

| **Profiles** | **Overall endometriosis & female only WHR** | | | | | | **Stage B endometriosis & female only WHR** | | | | | |
| --- | --- | --- | --- | --- | --- | --- | --- | --- | --- | --- | --- | --- |
|  | **OR** | **SE** | **z** | **P.Val** | **95% CI** | **R^2^** | **OR** | **SE** | **z** | **P.Val** | **95% CI** | **R^2^** |
| **Profile 1 (**P<0.01**)** | 1.030 | 0.060 | 0.50 | 0.614 | 0.918 - 1.156 | 0.0000 | 1.030 | 0.061 | 0.51 | 0.612 | 0.918 – 1.156 | 0.0000 |
| **Profile 2 (**P<0.05**)** | 1.045 | 0.142 | 0.32 | 0.746 | 0.800 - 1.364 | 0.0000 | 1.044 | 0.142 | 0.32 | 0.751 | 0.800 – 1.362 | 0.0000 |
| **Profile 3 (**P<0.10**)** | 0.982 | 0.196 | -0.09 | 0.926 | 0.663 - 1.453 | 0.0000 | 0.964 | 0.193 | -0.18 | 0.855 | 0.651 – 1.427 | 0.0000 |
| **Profile 4 (**P<0.20**)** | 0.974 | 0.286 | -0.09 | 0.930 | 0.548 - 1.732 | 0.0000 | 0.955 | 0.280 | -0.16 | 0.875 | 0.537 – 1.696 | 0.0000 |
| **Profile 5 (**P<0.30**)** | 0.818 | 0.306 | -0.54 | 0.590 | 0.393 - 1.700 | 0.0000 | 0.796 | 0.297 | -0.61 | 0.542 | 0.383 – 1.655 | 0.0000 |
| **Profile 6 (**P<0.40**)** | 0.813 | 0.362 | -0.46 | 0.642 | 0.340 - 1.945 | 0.0000 | 0.791 | 0.351 | -0.53 | 0.597 | 0.331 – 1.889 | 0.0000 |
| **Profile 7 (**P<0.50**)** | 0.876 | 0.455 | -0.25 | 0.800 | 0.317 - 2.423 | 0.0000 | 0.848 | 0.439 | -0.32 | 0.749 | 0.307 – 2.339 | 0.0000 |
| **Profile 8 (**P<0.75**)** | 1.189 | 0.813 | 0.25 | 0.800 | 0.311 - 4.542 | 0.0000 | 1.139 | 0.778 | 0.19 | 0.848 | 0.299 – 4.343 | 0.0000 |
| **Profile 9 (**P<1.00**)** | 1.327 | 1.094 | 0.34 | 0.731 | 0.264 - 6.675 | 0.0000 | 1.262 | 1.038 | 0.28 | 0.777 | 0.252 – 6.330 | 0.0000 |

**Supplementary Table 7.** Polygenic prediction results of overall BMI derived profile scores on overall endometriosis and stage B endometriosis.

| **Profiles** | **Overall endometriosis & overall BMI** | | | | | | **Stage B endometriosis & overall BMI** | | | | | |
| --- | --- | --- | --- | --- | --- | --- | --- | --- | --- | --- | --- | --- |
|  | **OR** | **SE** | **z** | **P.Val** | **95% CI** | **R^2^** | **OR** | **SE** | **z** | **P.Val** | **95% CI** | **R^2^** |
| **Profile 1 (**P<0.01**)** | 1.130 | 0.159 | 0.87 | 0.385 | 0.857 – 1.490 | 0.0001 | 1.134 | 0.159 | 0.89 | 0.373 | 0.860 – 1.494 | 0.0001 |
| **Profile 2 (**P<0.05**)** | 1.156 | 0.284 | 0.59 | 0.555 | 0.714 – 1.873 | 0.0000 | 1.164 | 0.286 | 0.62 | 0.536 | 0.719 – 1.885 | 0.0000 |
| **Profile 3 (**P<0.10**)** | 1.351 | 0.479 | 0.85 | 0.397 | 0.673 – 2.709 | 0.0001 | 1.355 | 0.481 | 0.86 | 0.392 | 0.676 – 2.716 | 0.0001 |
| **Profile 4 (**P<0.20**)** | 1.048 | 0.545 | 0.09 | 0.929 | 0.378 – 2.907 | 0.0000 | 1.044 | 0.543 | 0.08 | 0.934 | 0.377 – 2.894 | 0.0000 |
| **Profile 5 (**P<0.30**)** | 1.114 | 0.756 | 0.16 | 0.873 | 0.295 – 4.213 | 0.0000 | 1.106 | 0.749 | 0.15 | 0.882 | 0.293 – 4.175 | 0.0000 |
| **Profile 6 (**P<0.40**)** | 1.241 | 1.019 | 0.26 | 0.793 | 0.248 – 6.209 | 0.0000 | 1.213 | 0.996 | 0.24 | 0.814 | 0.242 – 6.067 | 0.0000 |
| **Profile 7 (**P<0.50**)** | 1.586 | 1.522 | 0.48 | 0.631 | 0.242 – 10.41 | 0.0000 | 1.552 | 1.487 | 0.46 | 0.647 | 0.237 – 10.16 | 0.0000 |
| **Profile 8 (**P<0.75**)** | 1.767 | 2.301 | 0.44 | 0.662 | 0.138 – 22.69 | 0.0000 | 1.702 | 2.214 | 0.41 | 0.683 | 0.133 – 21.77 | 0.0000 |
| **Profile 9 (**P<1.00**)** | 2.121 | 3.379 | 0.47 | 0.637 | 0.093 – 48.17 | 0.0000 | 2.020 | 3.213 | 0.44 | 0.659 | 0.089 – 45.65 | 0.0000 |

**Supplementary Table 8.** Polygenic prediction results of female-limited BMI derived profile scores on overall endometriosis and stage B endometriosis.

| **Profiles** | **Overall endometriosis & female only BMI** | | | | | | **Stage B endometriosis & female only BMI** | | | | | |
| --- | --- | --- | --- | --- | --- | --- | --- | --- | --- | --- | --- | --- |
|  | **OR** | **SE** | **z** | **P.Val** | **95% CI** | **R^2^** | **OR** | **SE** | **z** | **P.Val** | **95% CI** | **R^2^** |
| **Profile 1 (**P<0.01**)** | 1.109 | 0.103 | 1.13 | 0.260 | 0.926 – 1.330 | 0.0001 | 1.112 | 0.102 | 1.15 | 0.250 | 0.928 – 1.332 | 0.0001 |
| **Profile 2 (**P<0.05**)** | 1.108 | 0.191 | 0.60 | 0.551 | 0.790 – 1.554 | 0.0000 | 1.106 | 0.191 | 0.58 | 0.559 | 0.789 – 1.550 | 0.0000 |
| **Profile 3 (**P<0.10**)** | 1.121 | 0.272 | 0.47 | 0.640 | 0.696 – 1.804 | 0.0000 | 1.114 | 0.270 | 0.44 | 0.657 | 0.962 – 1.792 | 0.0000 |
| **Profile 4 (**P<0.20**)** | 1.256 | 0.426 | 0.67 | 0.503 | 0.645 – 2.442 | 0.0000 | 1.242 | 0.421 | 0.64 | 0.523 | 0.639 – 2.413 | 0.0000 |
| **Profile 5 (**P<0.30**)** | 1.551 | 0.664 | 1.03 | 0.305 | 0.670 – 3.590 | 0.0001 | 1.526 | 0.653 | 0.99 | 0.323 | 0.660 – 3.529 | 0.0001 |
| **Profile 6 (**P<0.40**)** | 1.874 | 0.976 | 1.21 | 0.227 | 0.675 – 5.199 | 0.0001 | 1.844 | 0.959 | 1.18 | 0.239 | 0.666 – 5.109 | 0.0001 |
| **Profile 7 (**P<0.50**)** | 2.130 | 1.289 | 1.24 | 0.213 | 0.648 – 6.980 | 0.0001 | 2.105 | 1.274 | 1.23 | 0.219 | 0.642 – 6.895 | 0.0001 |
| **Profile 8 (**P<0.75**)** | 3.260 | 2.645 | 1.45 | 0.146 | 0.663 – 16.00 | 0.0002 | 3.185 | 2.583 | 1.43 | 0.153 | 0.650 – 15.61 | 0.0002 |
| **Profile 9 (**P<1.00**)** | 4.160 | 4.112 | 1.44 | 0.149 | 0.599 – 28.87 | 0.0002 | 4.088 | 4.034 | 1.43 | 0.154 | 0.591 – 28.27 | 0.0002 |

**Supplementary Table 9.** Directionality of effects of the shared SNPs (primary dataset p<1x10^-3^ and look-up dataset p<0.05) between endometriosis (all and stage B only) and BMI/WHRadjBMI GWAS results.

| **Analyses**  **Primary vs. Lookup dataset**  **(Total No. of enriched SNPs)** | **No. of SNPs with P<0.05 with concordant direction** | **No. of SNPs with P<0.05 with discordant direction** | **Binom test P.Value for direction*** |
| --- | --- | --- | --- |
|  | **Endometriosis vs. WHRadjBMI** | | |
| **AllEndo vs. WHR** (52 SNPs) | 26 | 26 | 1.000 |
| **StbEndo vs. WHR** (57 SNPs) | 29 | 28 | 1.000 |
| **WHR vs. AllEndo** (45 SNPs) | 26 | 19 | 0.371 |
| **WHR vs. StbEndo** (52 SNPs) | 25 | 27 | 0.890 |
|  | **Endometriosis vs. BMI** | | |
| **AllEndo vs. BMI** (29 SNPs) | 13 | 16 | 0.711 |
| **StbEndo vs. BMI** (34 SNPs) | 16 | 18 | 0.864 |
| **BMI vs. AllEndo** (41 SNPs) | 19 | 22 | 0.755 |
| **BMI vs. StbEndo** (46 SNPs) | 25 | 21 | 0.659 |

AllEndo = GWAS results including All endometriosis cases, StbEndo = GWAS results including only stage B endometriosis cases, WHR = WHRadjBMI GWAS results, BMI = BMI GWAS results. *Two-sided binomial test with P=0.5.

**Supplementary Table 10.** Directionality of effects of the shared SNPs (primary dataset p<1x10^-3^ and look-up dataset p<0.05) between endometriosis (all and stage B only) and female-limited BMI/WHRadjBMI GWAS results.

| **Analyses**  **Primary vs. Lookup dataset**  **(Total No. of enriched SNPs)** | **No. of SNPs with P<0.05 with concordant direction** | **No. of SNPs with P<0.05 with discordant direction** | **Binom test P.Value for direction*** |
| --- | --- | --- | --- |
|  | **Endometriosis vs. WHRadjBMI** | | |
| **AllEndo vs. WHR** (50 SNPs) | 29 | 21 | 0.322 |
| **StbEndo vs. WHR** (47 SNPs) | 25 | 22 | 0.771 |
| **WHR vs. AllEndo** (57 SNPs) | 31 | 26 | 0.597 |
| **WHR vs. StbEndo** (53 SNPs) | 24 | 29 | 0.583 |
|  | **Endometriosis vs. BMI** | | |
| **AllEndo vs. BMI** (25 SNPs) | 12 | 13 | 1.000 |
| **StbEndo vs. BMI** (41 SNPs) | 22 | 19 | 0.755 |
| **BMI vs. AllEndo** (40 SNPs) | 17 | 23 | 0.430 |
| **BMI vs. StbEndo** (40 SNPs) | 18 | 22 | 0.636 |

AllEndo = GWAS results including all endometriosis cases, StbEndo = GWAS results including only stage B endometriosis cases, WHR = Female-limited WHRadjBMI GWAS results, BMI = Female-limited BMI GWAS results. *Two-sided binomial test with P=0.5.

**Supplementary Table 11.** Annotation of genetic enrichment analysis results between all endometriosis and WHRadjBMI (n=52).

| **SNP** | **Chr** | **Location** | | **Allele** | **Endometriosis** | | **Overall WHRadjBMI** | | | **Nearest** | **Distance** |
| --- | --- | --- | --- | --- | --- | --- | --- | --- | --- | --- | --- |
|  |  | **(B36)** | **(RAF)** | | **P.Value** | **OR (95% CIs)** | **P.Value** | **Effect** | **Std Err** | **Gene** |  |
| rs560584 | 1 | 168357136 | T(0.41) | | 1.42x10^-4^ | 1.14 (1.07-1.22) | 1.47x10^-5^ | -0.021 | 0.0046 | *KIFAP3* | 46632 |
| rs12700667 | 7 | 25868164 | A(0.74) | | 5.13x10^-7^ | 1.22 (1.13-1.32) | 4.39x10^-5^ | -0.023 | 0.0053 | *NFE2L3* | 290221 |
| rs2921188 | 3 | 12387115 | A(0.64) | | 5.92x10^-4^ | 1.13 (1.05-1.21) | 0.0011 | 0.017 | 0.0048 | *PPARG* | 0 |
| rs1250248 | 2 | 215995338 | A(0.27) | | 1.62x10^-5^ | 1.17 (1.09-1.26) | 0.0012 | 0.018 | 0.0052 | *FN1* | 0 |
| rs2630787 | 3 | 21847339 | C(0.52) | | 9.17x10^-4^ | 1.12 (1.05-1.19) | 0.0019 | -0.015 | 0.0045 | *ZNF659* | 79518 |
| rs1430788 | 2 | 67721916 | C(0.31) | | 9.35x10^-5^ | 1.15 (1.07-1.23) | 0.0027 | 0.016 | 0.0050 | *ETAA1* | 230878 |
| rs906721 | 3 | 184687691 | A(0.41) | | 6.10x10^-5^ | 1.16 (1.08-1.24) | 0.0042 | 0.015 | 0.0050 | *KLHL6* | 322 |
| rs1868894 | 4 | 187606728 | C(0.80) | | 2.33x10^-4^ | 1.16 (1.07-1.26) | 0.0049 | -0.018 | 0.0060 | *MTNR1A* | 85075 |
| rs3820282 | 1 | 22340802 | T(0.16) | | 3.36x10^-7^ | 1.26 (1.15-1.37) | 0.0050 | -0.019 | 0.0065 | *WNT4* | 0 |
| rs11101628 | 10 | 134856671 | G(0.05) | | 8.39x10^-4^ | 1.43 (1.16-1.77) | 0.0064 | -0.04 | 0.0140 | *KNDC1* | 0 |
| rs10510254 | 3 | 3254498 | C(0.08) | | 6.97x10^-4^ | 1.24 (1.09-1.40) | 0.0080 | 0.026 | 0.0095 | *CRBN* | 58107 |
| rs7914078 | 10 | 115067194 | T(0.65) | | 4.85x10^-4^ | 1.13 (1.06-1.21) | 0.0082 | -0.013 | 0.0048 | *TCF7L2* | 151133 |
| rs12681079 | 8 | 11521279 | C(0.26) | | 2.82x10^-4^ | 1.16 (1.07-1.25) | 0.0091 | -0.017 | 0.0064 | *GATA4* | 50598 |
| rs6687139 | 1 | 168439886 | A(0.90) | | 3.68x10^-4^ | 1.22 (1.09-1.36) | 0.0096 | -0.021 | 0.0077 | *KIFAP3* | 129382 |
| rs2527917 | 7 | 99405844 | C(0.52) | | 6.32x10^-4^ | 1.12 (1.05-1.19) | 0.011 | -0.012 | 0.0046 | *AZGP1* | 0 |
| rs6961094 | 7 | 101333036 | T(0.12) | | 2.23x10^-5^ | 1.25 (1.12-1.38) | 0.014 | -0.019 | 0.0076 | *CUTL1* | 0 |
| rs1035942 | 19 | 7150803 | A(0.28) | | 2.36x10^-4^ | 1.14 (1.07-1.24) | 0.014 | 0.013 | 0.0052 | *INSR* | 0 |
| rs12454262 | 18 | 46416077 | G(0.29) | | 5.07x10^-4^ | 1.13 (1.05-1.22) | 0.014 | -0.048 | 0.0185 | *MAPK4* | 0 |
| rs2480211 | 6 | 84795899 | T(0.27) | | 8.74x10^-4^ | 1.14 (1.05-1.23) | 0.014 | 0.013 | 0.0050 | *C6orf117* | 4240 |
| rs3856226 | 1 | 91017697 | C(0.55) | | 6.70x10^-4^ | 1.12 (1.05-1.20) | 0.015 | 0.012 | 0.0046 | *BARHL2* | 62314 |
| rs12820906 | 12 | 122059076 | A(0.77) | | 9.66x10^-4^ | 1.14 (1.06-1.24) | 0.015 | 0.014 | 0.0055 | *PITPNM2* | 0 |
| rs4695001 | 4 | 44004023 | T(0.15) | | 2.75x10^-4^ | 1.18 (1.08-1.29) | 0.016 | 0.016 | 0.0063 | *KCTD8* | 0 |
| rs10095984 | 8 | 131257447 | T(0.90) | | 5.28x10^-4^ | 1.23 (1.09-1.38) | 0.016 | 0.021 | 0.0084 | *DDEF1* | 0 |
| rs7017767 | 8 | 130534225 | C(0.91) | | 4.97x10^-4^ | 1.23 (1.09-1.38) | 0.017 | 0.022 | 0.0086 | *CCDC26* | 99816 |
| rs2107654 | 17 | 61063535 | C(0.47) | | 1.91x10^-6^ | 1.17 (1.09-1.25) | 0.020 | -0.011 | 0.0046 | *CCDC46* | 0 |
| rs1448792 | 9 | 22631633 | G(0.75) | | 4.60x10^-4^ | 1.14 (1.06-1.23) | 0.020 | 0.013 | 0.0053 | *AX747623* | 4566 |
| rs1898605 | 2 | 12509146 | T(0.70) | | 5.37x10^-4^ | 1.13 (1.05-1.22) | 0.020 | 0.012 | 0.0050 | *TRIB2* | 265513 |
| rs4862535 | 4 | 186634345 | A(0.83) | | 5.78x10^-4^ | 1.17 (1.07-1.28) | 0.022 | 0.022 | 0.0094 | *CCDC110* | 4468 |
| rs12310837 | 12 | 119364955 | G(0.16) | | 2.02x10^-4^ | 1.19 (1.08-1.30) | 0.023 | -0.014 | 0.0060 | *TRIAP1* | 1192 |
| rs1045738 | 17 | 4119692 | A(0.67) | | 4.04x10^-4^ | 1.13 (1.06-1.22) | 0.024 | -0.011 | 0.0048 | *UBE2G1* | 0 |
| rs17227503 | 2 | 154965619 | A(0.79) | | 8.58x10^-5^ | 1.18 (1.08-1.27) | 0.027 | -0.013 | 0.0057 | *GALNT13* | 0 |
| rs960792 | 2 | 204457495 | C(0.49) | | 2.10x10^-4^ | 1.13 (1.06-1.21) | 0.027 | 0.011 | 0.0046 | *CTLA4* | 10566 |
| rs6549200 | 3 | 69455764 | G(0.08) | | 3.43x10^-4^ | 1.24 (1.10-1.39) | 0.027 | -0.019 | 0.0081 | *FRMD4B* | 0 |
| rs4017074 | 3 | 134743117 | C(0.65) | | 5.09x10^-4^ | 1.13 (1.05-1.20) | 0.027 | -0.011 | 0.0048 | *CDV3* | 32147 |
| rs1795293 | 3 | 117761199 | G(0.14) | | 2.04x10^-4^ | 1.20 (1.09-1.32) | 0.028 | -0.015 | 0.0067 | *LSAMP* | 114130 |
| rs12433219 | 14 | 85760647 | C(0.51) | | 3.07x10^-4^ | 1.13 (1.06-1.20) | 0.028 | 0.011 | 0.0046 | *FLRT2* | 596623 |
| rs6860299 | 5 | 26471819 | A(0.11) | | 5.86x10^-4^ | 1.20 (1.08-1.33) | 0.030 | 0.017 | 0.0076 | *CDH9* | 444647 |
| rs17431067 | 20 | 8227734 | T(0.82) | | 2.74x10^-4^ | 1.18 (1.08-1.30) | 0.031 | -0.015 | 0.0067 | *PLCB1* | 0 |
| rs10854130 | 19 | 14311282 | A(0.60) | | 7.20x10^-4^ | 1.12 (1.05-1.20) | 0.033 | 0.010 | 0.0046 | *CD97* | 41931 |
| rs178642 | 14 | 22926736 | A(0.52) | | 8.90x10^-4^ | 1.14 (1.06-1.23) | 0.034 | -0.014 | 0.0064 | *MYH6* | 0 |
| rs701160 | 1 | 230159104 | C(0.80) | | 3.97x10^-4^ | 1.21 (1.09-1.35) | 0.038 | 0.015 | 0.0071 | *DISC1* | 0 |
| rs7291756 | 22 | 28656102 | A(0.75) | | 3.23x10^-4^ | 1.15 (1.06-1.24) | 0.039 | -0.011 | 0.0052 | *MTMR3* | 0 |
| rs2248556 | 1 | 69468286 | A(0.08) | | 2.83x10^-4^ | 1.25 (1.11-1.41) | 0.040 | -0.019 | 0.0089 | RP4-686N16.1 | 337170 |
| rs9358819 | 6 | 25120271 | A(0.09) | | 5.39x10^-4^ | 1.22 (1.09-1.37) | 0.040 | 0.017 | 0.0077 | NR_000030 | 34876 |
| rs11070352 | 15 | 39945008 | G(0.33) | | 8.18x10^-4^ | 1.12 (1.05-1.20) | 0.040 | 0.011 | 0.0052 | *SPTBN5* | 0 |
| rs3734431 | 6 | 52951388 | G(0.08) | | 5.75x10^-4^ | 1.25 (1.10-1.41) | 0.043 | 0.018 | 0.0084 | *GSTA4* | 0 |
| rs4685929 | 3 | 5424157 | G(0.77) | | 6.66x10^-4^ | 1.14 (1.06-1.24) | 0.043 | 0.012 | 0.0057 | *EDEM1* | 187514 |
| rs2918300 | 19 | 8648027 | T(0.36) | | 6.39x10^-4^ | 1.13 (1.05-1.20) | 0.044 | -0.011 | 0.0050 | *MGC33407* | 20726 |
| rs1154245 | 18 | 19798212 | G(0.79) | | 5.48x10^-4^ | 1.15 (1.06-1.25) | 0.045 | 0.012 | 0.0057 | *LAMA3* | 9184 |
| rs9667927 | 11 | 120127835 | C(0.07) | | 8.66x10^-4^ | 1.31 (1.12-1.53) | 0.046 | 0.030 | 0.0142 | *GRIK4* | 0 |
| rs2037664 | 2 | 154987069 | T(0.93) | | 1.90x10^-4^ | 1.29 (1.13-1.48) | 0.047 | -0.020 | 0.0094 | *GALNT13* | 0 |
| rs17626899 | 15 | 92016929 | G(0.42) | | 2.23x10^-4^ | 1.13 (1.06-1.21) | 0.048 | -0.010 | 0.0048 | *DQ601182* | 246337 |

**Supplementary Table 12.** Annotation of genetic enrichment analysis results between stage B endometriosis and WHRadjBMI (n=57).

| **SNP** | **Chr** | | **Location** | **Allele** | | **Stage B Endometriosis** | | **Overall WHRadjBMI** | | | **Nearest** | **Distance** |
| --- | --- | --- | --- | --- | --- | --- | --- | --- | --- | --- | --- | --- |
|  |  | **(B36)** | | | **(RAF)** | **P.Value** | **OR (95% C.I.)** | **P.Value** | **Effect** | **Std Err** | **Gene** |  |
| rs11619804 | 13 | 49888131 | | | C(0.53) | 4.88x10^-4^ | 1.17(1.07-1.28) | 1.06x10^-5^ | 0.022 | 0.0048 | *CAB39L* | 0 |
| rs12700667 | 7 | 25868164 | | | A(0.74) | 3.34x10^-9^ | 1.36(1.23-1.50) | 4.39x10^-5^ | -0.023 | 0.0053 | *NFE2L3* | 290221 |
| rs2782659 | 6 | 45794768 | | | G(0.33) | 4.24x10^-4^ | 1.18 (1.08-1.30) | 9.20x10^-5^ | 0.02 | 0.0050 | *RUNX2* | 167970 |
| rs6556301 | 5 | 176460183 | | | G(0.63) | 7.39x10^-4^ | 1.17 (1.07-1.28) | 1.90x10^-4^ | -0.021 | 0.0053 | *FGFR4* | 2450 |
| rs1250248 | 2 | 215995338 | | | A(0.27) | 2.93x10^-8^ | 1.32 (1.19-1.45) | 0.0012 | 0.018 | 0.0052 | *FN1* | 0 |
| rs4131816 | 1 | 161662648 | | | T(0.85) | 5.41x10^-4^ | 1.24 (1.10-1.41) | 0.0015 | 0.022 | 0.0067 | *NUF2* | 70470 |
| rs9912335 | 17 | 77552948 | | | T(0.69) | 3.09x10^-4^ | 1.19 (1.08-1.31) | 0.0035 | -0.021 | 0.0070 | *ASPSCR1* | 0 |
| rs10878362 | 12 | 64703760 | | | C(0.69) | 4.92x10^-4^ | 1.19 (1.08-1.31) | 0.0036 | 0.015 | 0.0050 | *HMGA2* | 57421 |
| rs2807357 | 1 | 22364571 | | | A(0.64) | 9.66x10^-4^ | 1.16 (1.06-1.27) | 0.0037 | -0.015 | 0.0050 | *WNT4* | 22373 |
| rs906721 | 3 | 184687691 | | | A(0.41) | 1.41x10^-4^ | 1.20 (1.09-1.32) | 0.0042 | 0.015 | 0.0050 | *KLHL6* | 322 |
| rs12267660 | 10 | 4419530 | | | G(0.85) | 7.98x10^-4^ | 1.24 (1.09-1.40) | 0.0046 | 0.02 | 0.0069 | *CR749391* | 191913 |
| rs11685481 | 2 | 67590253 | | | C(0.15) | 8.36x10^-4^ | 1.23 (1.09-1.38) | 0.0048 | 0.018 | 0.0060 | *ETAA1* | 99215 |
| rs3820282 | 1 | 22340802 | | | T(0.16) | 5.21x10^-7^ | 1.35 (1.20-1.52) | 0.0050 | -0.019 | 0.0065 | *WNT4* | 0 |
| rs16890431 | 4 | 14040485 | | | G(0.05) | 1.07x10^-4^ | 1.49 (1.22-1.83) | 0.0060 | -0.029 | 0.0102 | *MGC4836* | 42291 |
| rs16972667 | 15 | 79118422 | | | G(0.05) | 1.22x10^-5^ | 2.11 (1.51-2.95) | 0.0065 | -0.039 | 0.0135 | *IL16* | 35021 |
| rs498439 | 1 | 115795199 | | | T(0.17) | 2.07x10^-5^ | 1.31(1.15-1.48) | 0.0065 | -0.065 | 0.0227 | *NGFB* | 112818 |
| rs11144059 | 9 | 70671417 | | | G(0.15) | 7.68x10^-4^ | 1.23 (1.09-1.39) | 0.0066 | 0.069 | 0.0242 | *PIP5K1B* | 0 |
| rs7792269 | 7 | 47679838 | | | C(0.43) | 5.96x10^-4^ | 1.16 (1.07-1.27) | 0.0072 | 0.013 | 0.0047 | *MGC16075* | 87761 |
| rs2408141 | 12 | 43789830 | | | C(0.57) | 8.91x10^-5^ | 1.19 (1.09-1.30) | 0.0080 | 0.013 | 0.0046 | *FKSG42* | 44368 |
| rs10510254 | 3 | 3254498 | | | C(0.07) | 1.86x10^-4^ | 1.36 (1.16-1.61) | 0.0080 | 0.026 | 0.0095 | *CRBN* | 58107 |
| rs760923 | 1 | 22229804 | | | T(0.76) | 7.37x10^-4^ | 1.19 (1.07-1.32) | 0.0086 | -0.015 | 0.0054 | *ELA3A* | 18181 |
| rs1479605 | 10 | 115084799 | | | A(0.64) | 5.67x10^-4^ | 1.17 (1.07-1.28) | 0.0098 | -0.013 | 0.0047 | *TCF7L2* | 168738 |
| rs2527917 | 7 | 99405844 | | | C(0.51) | 1.31x10^-4^ | 1.18 (1.09-1.29) | 0.0110 | -0.012 | 0.0046 | *AZGP1* | 0 |
| rs6668131 | 1 | 10575192 | | | G(0.10) | 6.06x10^-4^ | 1.30 (1.12-1.52) | 0.0120 | -0.021 | 0.0081 | *PEX14* | 0 |
| rs6961094 | 7 | 101333036 | | | T(0.12) | 1.16x10^-5^ | 1.36 (1.19-1.56) | 0.0140 | -0.019 | 0.0076 | *CUTL1* | 0 |
| rs12454262 | 18 | 46416077 | | | G(0.29) | 5.84x10^-4^ | 1.18 (1.07-1.30) | 0.0140 | -0.048 | 0.0185 | *MAPK4* | 0 |
| rs9536500 | 13 | 53124625 | | | G(0.88) | 9.46x10^-4^ | 1.27 (1.10-1.47) | 0.0150 | 0.021 | 0.0084 | *OLFM4* | 600435 |
| rs6711230 | 2 | 73996236 | | | C(0.05) | 5.72x10^-4^ | 1.93 (1.33-2.81) | 0.0160 | 0.052 | 0.0206 | *ACTG2* | 0 |
| rs17178718 | 17 | 5492414 | | | T(0.86) | 8.12x10^-4^ | 1.24 (1.09-1.41) | 0.0170 | -0.019 | 0.0074 | *NLRP1* | 63857 |
| rs13335480 | 16 | 61624033 | | | A(0.14) | 4.60x10^-4^ | 1.25 (1.10-1.41) | 0.0200 | 0.016 | 0.0066 | *CDH8* | 996495 |
| rs822531 | 7 | 148260692 | | | T(0.79) | 9.40x10^-4^ | 1.20 (1.08-1.33) | 0.0200 | 0.016 | 0.0064 | *EZH2* | 8319 |
| rs9999033 | 4 | 186628317 | | | G(0.83) | 3.48x10^-4^ | 1.23 (1.10-1.38) | 0.0220 | 0.022 | 0.0091 | *CCDC110* | 0 |
| rs224816 | 5 | 81697753 | | | G(0.48) | 8.04x10^-4^ | 1.16 (1.06-1.26) | 0.0220 | 0.011 | 0.0046 | *RPS23* | 87761 |
| rs2100807 | 3 | 117506680 | | | C(0.18) | 7.82x10^-4^ | 1.21 (1.08-1.35) | 0.0240 | -0.015 | 0.0061 | *LSAMP* | 0 |
| rs12097741 | 1 | 67300594 | | | C(0.18) | 8.30x10^-4^ | 1.21 (1.08-1.35) | 0.0240 | -0.015 | 0.0064 | *SLC35D1* | 8277 |
| rs1535249 | 20 | 19555534 | | | A(0.05) | 6.58x10^-4^ | 2.28 (1.42-3.67) | 0.0260 | 0.055 | 0.0234 | *SLC24A3* | 0 |
| rs6431897 | 2 | 7851260 | | | C(0.79) | 7.99x10^-5^ | 1.23 (1.11-1.37) | 0.0310 | 0.013 | 0.0058 | *C2orf46* | 413464 |
| rs1568248 | 3 | 117796042 | | | C(0.10) | 2.59x10^-4^ | 1.30 (1.13-1.51) | 0.0310 | -0.018 | 0.0078 | *LSAMP* | 148973 |
| rs13092529 | 3 | 21009923 | | | G(0.89) | 4.03x10^-4^ | 1.30 (1.12-1.49) | 0.0310 | 0.017 | 0.0073 | *HPX-42* | 412299 |
| rs858031 | 21 | 38023983 | | | C(0.95) | 4.88x10^-4^ | 1.59 (1.22-2.06) | 0.0330 | 0.03 | 0.0135 | *KCNJ6* | 0 |
| rs2911890 | 4 | 37472706 | | | G(0.88) | 2.18x10^-4^ | 1.29 (1.13-1.49) | 0.0340 | -0.015 | 0.0069 | *PGM2* | 32031 |
| rs416190 | 1 | 114599221 | | | A(0.21) | 8.08x10^-4^ | 1.20 (1.08-1.33) | 0.0350 | 0.012 | 0.0056 | *SYT6* | 101225 |
| rs9890598 | 17 | 27061309 | | | C(0.91) | 3.24x10^-4^ | 1.33 (1.14-1.55) | 0.0360 | -0.018 | 0.0083 | *C17orf79* | 141703 |
| rs2048740 | 2 | 166020802 | | | C(0.08) | 8.47x10^-4^ | 1.32 (1.12-1.55) | 0.0360 | 0.019 | 0.0086 | *FAM130A2* | 13601 |
| rs7637969 | 3 | 192465170 | | | C(0.22) | 1.19x10^-4^ | 1.23 (1.11-1.36) | 0.0370 | -0.012 | 0.0057 | *UTS2D* | 2875 |
| rs11060440 | 12 | 128611376 | | | T(0.10) | 1.74x10^-4^ | 1.45 (1.19-1.76) | 0.0380 | 0.021 | 0.0095 | *TMEM132D* | 0 |
| rs3016818 | 18 | 569716 | | | C(0.71) | 9.88x10^-4^ | 1.18 (1.07-1.31) | 0.0380 | 0.011 | 0.0051 | *CETN1* | 653 |
| rs2241490 | 11 | 121475990 | | | T(0.27) | 9.70x10^-4^ | 1.20 (1.07-1.34) | 0.0390 | -0.011 | 0.0053 | *BRCC2* | 15282 |
| rs4082225 | 3 | 3283886 | | | T(0.05) | 1.70x10^-5^ | 1.65 (1.31-2.08) | 0.0410 | 0.034 | 0.0159 | *CRBN* | 87495 |
| rs529439 | 5 | 6669277 | | | A(0.69) | 8.71x10^-4^ | 1.17 (1.07-1.28) | 0.0420 | -0.089 | 0.0422 | *NSUN2* | 0 |
| rs10840415 | 11 | 10421488 | | | G(0.87) | 7.51x10^-4^ | 1.24 (1.10-1.41) | 0.0430 | 0.014 | 0.0067 | *AMPD3* | 7312 |
| rs2509963 | 11 | 61949507 | | | C(0.74) | 7.99x10^-4^ | 1.18 (1.07-1.30) | 0.0430 | 0.011 | 0.0053 | *SCGB1A1* | 2263 |
| rs11857306 | 15 | 54533295 | | | C(0.38) | 7.19x10^-5^ | 1.20 (1.10-1.31) | 0.0440 | -0.001 | 0.0047 | *MNS1* | 0 |
| rs6923006 | 6 | 5387973 | | | G(0.06) | 8.75x10^-5^ | 1.47 (1.21-1.78) | 0.0440 | -0.02 | 0.0096 | *FARS2* | 0 |
| rs4791142 | 17 | 61089647 | | | A(0.44) | 1.99x10^-4^ | 1.18 (1.08-1.28) | 0.0460 | -0.009 | 0.0046 | *CCDC46* | 0 |
| rs6544800 | 2 | 45373126 | | | G(0.07) | 1.18x10^-4^ | 1.44 (1.19-1.74) | 0.0470 | 0.022 | 0.0104 | *UNQ6975* | 37541 |
| rs7602149 | 2 | 114357038 | | | C(0.08) | 3.23x10^-4^ | 1.35 (1.15-1.59) | 0.0470 | -0.018 | 0.0089 | *ACTR3* | 6969 |

**Supplementary Table 13.** Results of the Mammalian Gene Expression Uterus database (MGEx-Udb)([4](#_ENREF_3)) look up for the 16 genes in human endometrium tissue from individuals with endometriosis and without.

| **SNP** | **Chr** | **Position** | **Distance** | **Closest gene** | **Normal endometrium tissue** | **Endometriosis endometrium tissue** |
| --- | --- | --- | --- | --- | --- | --- |
| rs560584 | 1 | 168357136 | 46632 | *KIFAP3* | Transcribed | Transcribed |
| rs11619804 | 13 | 49888131 | 0 | *CAB39L* | Transcribed | Transcribed |
| rs12700667 | 7 | 25868164 | 290221 | *NFE2L3* | Transcribed | Transcribed |
| ***rs709156*** | ***3*** | ***12436615*** | ***0*** | ***PPARG*** | ***Dormant*** | ***Transcribed*** |
| rs1250248 | 2 | 215995338 | 0 | *FN1* | Transcribed | Transcribed |
| rs1356100 | 3 | 21863481 | 95660 | *ZNF659* | Dormant | Dormant |
| rs2860517 | 2 | 67603178 | 112140 | *ETAA1* | Transcribed | Transcribed |
| rs906721 | 3 | 184687691 | 322 | *KLHL6* | Transcribed | Transcribed |
| rs1868894 | 4 | 187606728 | 85075 | *MTNR1A* | Dormant | Dormant |
| rs3820282 | 1 | 22340802 | 0 | *WNT4* | Transcribed | Transcribed |
| rs2782666 | 6 | 45799605 | 172807 | *RUNX2* | Transcribed | Transcribed |
| ***rs6556301*** | ***5*** | ***176460183*** | ***2450*** | ***FGFR4*** | ***Dormant*** | ***Transcribed*** |
| ***rs4131816*** | ***1*** | ***161662648*** | ***70470*** | ***NUF2*** | ***Transcribed*** | ***Dormant*** |
| rs7326191 | 13 | 48737980 | 0 | *CDADC1* | Transcribed | Transcribed |
| rs9912335 | 17 | 77552948 | 0 | *ASPSCR1* | Dormant | Dormant |
| rs10878362 | 12 | 64703760 | 57421 | *HMGA2* | Transcribed | Transcribed |

**Supplementary Table 14.** Results of the *cis*-eQTL analysis in the MUTHER data for abdominal fat expression of the 16 genes([5](#_ENREF_4)).

| **SNP** | **Chr** | **Position** | **Closest Gene** | **Beta** | **P.Value** | **Adjusted P.Value**** |
| --- | --- | --- | --- | --- | --- | --- |
| rs560584 | 1 | 168357136 | *KIFAP3* | -0.015 | 0.07 | 0.79 |
| rs11619804 | 13 | 49888131 | *CAB39L* | 0.006 | 0.31 | 0.92 |
| rs12700667 | 7 | 25868164 | *HNRNPA2B1** | 0.024 | 0.52 | 0.98 |
| rs709156 | 3 | 12436615 | *PPARG* | -0.029 | 0.15 | 0.93 |
| rs1250248 | 2 | 215995338 | *FN1* | 0.003 | 0.57 | 0.98 |
| rs1356100 | 3 | 21863481 | *N/A** | N/A | N/A | N/A |
| rs2860517 | 2 | 67603178 | *ETAA1* | -0.002 | 0.7 | 0.98 |
| rs906721 | 3 | 184687691 | *KLHL24** | -0.032 | 0.26 | 0.95 |
| rs1868894 | 4 | 187606728 | *MTNR1A* | -0.008 | 0.21 | 0.94 |
| rs3820282 | 1 | 22340802 | *WNT4* | -0.007 | 0.37 | 0.96 |
| rs2782666 | 6 | 45799605 | *CLIC5** | 0.033 | 0.07 | 0.79 |
| rs6556301 | 5 | 176460183 | *FGFR4* | -0.002 | 0.7 | 0.98 |
| rs4131816 | 1 | 161662648 | *NUF2* | 0.001 | 0.85 | 0.99 |
| rs7326191 | 13 | 48737980 | *CDADC1* | -0.003 | 0.63 | 0.98 |
| rs9912335 | 17 | 77552948 | *ASPSCR1* | -0.01 | 0.09 | 0.82 |
| rs10878362 | 12 | 64703760 | *HMGA2* | -0.011 | 0.05 | 0.71 |

* No probe covering *NFE2L3, ZNF659, KHL6 and RUNX2 respectively.* Where, there was a probe covering another close by gene within 500kb on either side of SNP, the results for those genes are reported.

****** Benjamin Hochberg corrected p-values.

**Supplementary Table 15.** Results of the differential *cis*-eQTL analysis between abdominal and gluteal fat in the MolOBB data on the 16 genes.

| **SNP** | **Chr** | **Position** | **Closest gene** | **Distance** | **Fold change** | **P.Value** | **Adjusted P.Value**** |
| --- | --- | --- | --- | --- | --- | --- | --- |
| **rs560584** | **1** | **168357136** | ***KIFAP3*** | **36163** | **0.14** | **6.70x10^-3^** | **0.04** |
| rs11619804 | 13 | 49888131 | *CAB39L** | *N/A* | *N/A* | *N/A* | *N/A* |
| rs12700667 | 7 | 25868164 | *HNRNPA2B1** | 327908 | 0.04 | 0.35 | 0.56 |
| rs709156 | 3 | 12436615 | *PPARG* | 0 | 0.15 | 0.15 | 0.32 |
| rs1250248 | 2 | 215995338 | *FN1* | 0 | 0.16 | 0.04 | 0.15 |
| rs1356100 | 3 | 21863481 | *N/A** | N/A | N/A | N/A | N/A |
| rs2860517 | 2 | 67603178 | *ETAA1* | 111997 | -0.17 | 7.31x10^-3^ | 0.04 |
| rs906721 | 3 | 184687691 | *KLHL24** | 148359 | -0.13 | 0.07 | 0.19 |
| rs1868894 | 4 | 187606728 | *N/A** | N/A | N/A | N/A | N/A |
| rs3820282 | 1 | 22340802 | *HSPG2** | 204425 | -0.16 | 0.02 | 0.09 |
| rs2782666 | 6 | 45799605 | *CLIC5** | 157123 | 0.2 | 0.01 | 0.06 |
| *rs6556301* | *5* | *176460183* | *B4GALT7** | *499524* | *0.21* | *5.70x10^-4^* | *7.80x10^-3^* |
| rs4131816 | 1 | 161662648 | *N/A** | N/A | N/A | N/A | N/A |
| rs7326191 | 13 | 48737980 | *CDADC1* | 0 | 0.12 | 0.03 | 0.1 |
| rs9912335 | 17 | 77552948 | *ASPSCR1* | 0 | 0.14 | 0.26 | 0.47 |
| rs10878362 | 12 | 64703760 | *LLPH** | 99349 | -0.02 | 0.76 | 0.87 |

* No probe covering *CAB39L, NFE2L3, ZNF659, KHL6, MTNR1A,WNT4, RUNX2, FGFR4,NUF2 and HMGA2*. Where there was a probe covering another close by gene within 500kb on either side of SNP, the results for those genes are reported.

****** Genome-wide FDR corrected F test p-value.

**Supplementary Table 16.** Results of over/under-representation of (a) 88 genes associated with all endometriosis (P<1.0x10^-3^) and WHRadjBMI (P<0.05), and (b) 103 genes associated with stage B endometriosis (P<1.0x10^-3^) and WHRadjBMI (P<0.05), in 241 biological processes in PANTHER.

|  | **Number of genes in** | | |  |
| --- | --- | --- | --- | --- |
| **Biological process** | **Biological process** | **Associated regions** | | **P.Value** |
|  |  | **Observed** | **Expected** |  |
| **(a) Overall endometriosis vs. WHRadjBMI** |  |  |  |  |
| Cellular process | 6400 | 51 | 28.16 | 7.85x10^-7^ |
| Cell communication | 4504 | 40 | 19.82 | 2.09x10^-6^ |
| Developmental process | 3204 | 31 | 14.10 | 1.20x10^-5^ |
| Immune system process | 2716 | 22 | 11.95 | 4.27x10^-3^ |
| Cell adhesion | 1411 | 14 | 6.21 | 4.89x10^-3^ |
| System process | 2466 | 19 | 10.85 | 1.36x10^-2^ |
| Cell cycle | 1867 | 15 | 8.21 | 2.48x10^-2^ |
| Response to stimulus | 1921 | 14 | 8.45 | 6.60x10^-2^ |
| Reproduction | 1143 | 8 | 5.03 | 0.17 |
| Generation of precursor metabolites | 338 | 3 | 1.49 | 0.19 |
| Metabolic process | 8351 | 43 | 36.74 | 0.19 |
| Transport | 2984 | 17 | 13.13 | 0.23 |
| Cellular component organization | 1496 | 9 | 6.58 | 0.31 |
| Apoptosis | 1085 | 7 | 4.77 | 0.33 |
| **(b) Stage B endometriosis vs. WHRadjBMI** |  |  |  |  |
| Developmental process | 3204 | 32 | 16.50 | 1.25x10^-4^ |
| Cellular process | 6400 | 46 | 32.96 | 7.8x10^-3^ |
| Cell communication | 4504 | 34 | 23.20 | 1.29x10^-2^ |
| Cell cycle | 1867 | 17 | 9.62 | 1.71x10^-2^ |
| Transport | 2984 | 23 | 15.37 | 5.05x10^-2^ |
| Response to stimulus | 1921 | 16 | 9.89 | 6.20x10^-2^ |
| Immune system process | 2716 | 20 | 13.99 | 8.45x10^-2^ |
| System process | 2466 | 18 | 12.70 | 0.13 |
| Cellular component organization | 1496 | 10 | 7.70 | 0.35 |
| Metabolic process | 8351 | 46 | 43.01 | 0.55 |
| Localization | 160 | 1 | 0.82 | 0.56 |
| Apoptosis | 1085 | 6 | 5.59 | 0.83 |
| Cell adhesion | 1411 | 6 | 7.27 | 0.85 |
| Generation of precursor metabolites | 338 | 1 | 1.74 | 1 |

**Supplementary Table 17.** Results of over/under-representation of (a) 88 genes associated with all endometriosis (P<1.0x10^-3^) and WHRadjBMI (P<0.05), and (b) 103 genes associated with stage B endometriosis (P<1.0x10^-3^) and WHRadjBMI (P<0.05), in 176 curated biological pathways in PANTHER.

|  | **Number of genes in** | | |  |
| --- | --- | --- | --- | --- |
| **Pathways** | **Pathway** | **Associated regions** | | **P.Value** |
|  |  | **Observed** | **Expected** |  |
| **(a) Overall endometriosis vs. WHRadjBMI** |  |  |  |  |
| Wnt signaling pathway | 297 | 7 | 1.08 | 1.07x10^-4^ |
| Gonadotropin releasing hormone receptor pathway | 228 | 5 | 0.83 | 1.48x10^-3^ |
| Cadherin signaling pathway | 153 | 4 | 0.55 | 2.40x10^-3^ |
| Heterotrimeric G-protein signaling pathway-Gq alpha and Go alpha mediated pathway | 113 | 3 | 0.41 | 8.21x10^-3^ |
| Metabotropic glutamate receptor group III pathway | 56 | 2 | 0.2 | 1.78x10^-2^ |
| 2-arachidonoylglycerol biosynthesis | 6 | 1 | 0.02 | 2.15x10^-2^ |
| Integrin signalling pathway | 175 | 3 | 0.63 | 2.60x10^-2^ |
| Interleukin signaling pathway | 95 | 2 | 0.34 | 4.69x10^-2^ |
| Inflammation mediated by chemokine and cytokine signaling | 233 | 3 | 0.84 | 5.30x10^-2^ |
| Alzheimer disease-presenilin pathway | 109 | 2 | 0.39 | 5.98x10^-2^ |
| Endogenous_cannabinoid_signaling | 24 | 1 | 0.09 | 8.33x10^-2^ |
| Heterotrimeric G-protein signaling pathway-Gi alpha and Gs alpha mediated pathway | 134 | 2 | 0.49 | 8.54x10^-2^ |
| Alpha adrenergic receptor signaling pathway | 25 | 1 | 0.09 | 8.66x10^-2^ |
| Oxidative stress response | 28 | 1 | 0.1 | 9.65x10^-2^ |
| Histamine H1 receptor mediated signaling pathway | 29 | 1 | 0.11 | 9.98x10^-2^ |
| Angiogenesis | 152 | 2 | 0.55 | 0.11 |
| FAS signaling pathway | 31 | 1 | 0.11 | 0.11 |
| Insulin/IGF pathway-mitogen activated protein kinase kinase/MAP kinase cascade | 32 | 1 | 0.12 | 0.11 |
| Angiotensin II-stimulated signaling through G proteins and beta-arrestin | 37 | 1 | 0.13 | 0.13 |
| Insulin/IGF pathway-protein kinase B signaling cascade | 37 | 1 | 0.13 | 0.13 |
| Notch signaling pathway | 40 | 1 | 0.14 | 0.14 |
| Oxytocin receptor mediated signaling pathway | 43 | 1 | 0.16 | 0.14 |
| Thyrotropin-releasing hormone receptor signaling pathway | 45 | 1 | 0.16 | 0.15 |
| Blood coagulation | 45 | 1 | 0.16 | 0.15 |
| p53 pathway feedback loops 2 | 47 | 1 | 0.17 | 0.16 |
| PI3 kinase pathway | 47 | 1 | 0.17 | 0.16 |
| Ionotropic glutamate receptor pathway | 49 | 1 | 0.18 | 0.16 |
| 5HT2 type receptor mediated signaling pathway | 50 | 1 | 0.18 | 0.17 |
| Alzheimer disease-amyloid secretase pathway | 62 | 1 | 0.22 | 0.20 |
| Ubiquitin proteasome pathway | 62 | 1 | 0.22 | 0.20 |
| B cell activation | 64 | 1 | 0.23 | 0.21 |
| Nicotinic acetylcholine receptor signaling pathway | 74 | 1 | 0.27 | 0.24 |
| Endothelin signaling pathway | 79 | 1 | 0.29 | 0.25 |
| p53 pathway | 81 | 1 | 0.29 | 0.26 |
| Cytoskeletal regulation by Rho GTPase | 82 | 1 | 0.3 | 0.26 |
| Parkinson disease | 88 | 1 | 0.32 | 0.27 |
| FGF signaling pathway | 115 | 1 | 0.42 | 0.34 |
| PDGF signaling pathway | 132 | 1 | 0.48 | 0.38 |
| Huntington disease | 142 | 1 | 0.51 | 0.40 |
| **(b) Stage B endometriosis vs. WHRadjBMI** |  |  |  |  |
| Wnt signaling pathway | 297 | 7 | 1.32 | 3.84x10^-4^ |
| Cadherin signaling pathway | 153 | 5 | 0.68 | 6.42x10^-4^ |
| TGF-beta signaling pathway | 95 | 4 | 0.42 | 1.48x10^-3^ |
| FGF signaling pathway | 115 | 4 | 0.51 | 2.96x10^-3^ |
| Alzheimer disease-presenilin pathway | 109 | 3 | 0.48 | 1.30x10^-2^ |
| Gonadotropin releasing hormone receptor pathway | 228 | 4 | 1.01 | 1.92x10^-2^ |
| Inflammation mediated by chemokine and cytokine signaling | 233 | 4 | 1.04 | 2.06x10^-2^ |
| Purine metabolism | 7 | 1 | 0.03 | 3.07x10^-2^ |
| B cell activation | 64 | 2 | 0.28 | 3.34x10^-2^ |
| Circadian clock system | 9 | 1 | 0.04 | 3.93x10^-2^ |
| Cytoskeletal regulation by Rho GTPase | 82 | 2 | 0.36 | 5.21x10^-2^ |
| Interleukin signaling pathway | 95 | 2 | 0.42 | 6.74x10^-2^ |
| Hedgehog signaling pathway | 21 | 1 | 0.09 | 8.92x10^-2^ |
| Heterotrimeric G-protein signaling pathway-Gq alpha and Go alpha mediated pathway | 113 | 2 | 0.5 | 9.06x10^-2^ |
| Axon guidance mediated by Slit/Robo | 25 | 1 | 0.11 | 0.11 |
| Heterotrimeric G-protein signaling pathway-Gi alpha and Gs alpha mediated pathway | 134 | 2 | 0.6 | 0.12 |
| Synaptic_vesicle_trafficking | 29 | 1 | 0.13 | 0.12 |
| Histamine H1 receptor mediated signaling pathway | 29 | 1 | 0.13 | 0.12 |
| Huntington disease | 142 | 2 | 0.63 | 0.13 |
| Axon guidance mediated by netrin | 34 | 1 | 0.15 | 0.14 |
| Angiogenesis | 152 | 2 | 0.68 | 0.15 |
| Angiotensin II-stimulated signaling through G proteins and beta-arrestin | 37 | 1 | 0.16 | 0.15 |
| Notch signaling pathway | 40 | 1 | 0.18 | 0.16 |
| p38 MAPK pathway | 41 | 1 | 0.18 | 0.17 |
| Muscarinic acetylcholine receptor 2 and 4 signaling pathway | 43 | 1 | 0.19 | 0.17 |
| Integrin signalling pathway | 175 | 2 | 0.78 | 0.18 |
| Muscarinic acetylcholine receptor 1 and 3 signaling pathway | 46 | 1 | 0.2 | 0.19 |
| Ionotropic glutamate receptor pathway | 49 | 1 | 0.22 | 0.20 |
| Metabotropic glutamate receptor group III pathway | 56 | 1 | 0.25 | 0.22 |
| Alzheimer disease-amyloid secretase pathway | 62 | 1 | 0.28 | 0.24 |
| Ras Pathway | 73 | 1 | 0.32 | 0.28 |
| Nicotinic acetylcholine receptor signaling pathway | 74 | 1 | 0.33 | 0.28 |
| Endothelin signaling pathway | 79 | 1 | 0.35 | 0.30 |
| T cell activation | 82 | 1 | 0.36 | 0.31 |
| Parkinson disease | 88 | 1 | 0.39 | 0.32 |
| PDGF signaling pathway | 132 | 1 | 0.59 | 0.45 |

**Supplementary Table 18.** Sensitivity analysis on over/under-representation of genes associated with all endometriosis and WHRadjBMI with using two additional p-value thresholds: (1) P<1x10^-4^ and (2) P<1x10^-2^ in comparison to our original enrichment threshold of p<1x10^-3^, in 176 curated biological pathways in PANTHER.

| **Pathways:**  **Original enrichment threshold (P<1x10^-3^)** | **N of genes in** | | | **P-value** | **Pathways:**  **Original enrichment threshold (P<1x10^-4^)** | **N genes in** | | **P-value** | **Pathways:**  **Original enrichment threshold (P<1x10^-2^)** | **N genes in** | | **P-value** |
| --- | --- | --- | --- | --- | --- | --- | --- | --- | --- | --- | --- | --- |
|  | **Pathway** | **Associated regions** | |  |  | **associated regions** | |  |  | **associated regions** | |  |
|  |  | **Obs** | **Exp** |  |  | **Obs** | **Exp** |  |  | **Obs** | **Exp** |  |
| Wnt signaling pathway | 297 | 7 | 1.08 | 1.07x10^-4^ | Alzheimer disease-presenilin pathway | 1 | 0.07 | 6.78x10^-2^ | Wnt signaling pathway | 22 | 4.89 | 8.29x10^-9^ |
| Gonadotropin releasing hormone receptor p^*^ | 228 | 5 | 0.83 | 1.48x10^-3^ | Cadherin signaling | 1 | 0.1 | 9.39x10^-2^ | Cadherin signaling | 14 | 2.52 | 3.86x10^-7^ |
| Cadherin signaling | 153 | 4 | 0.55 | 2.40x10^-3^ | Integrin signalling | 1 | 0.11 | 0.11 | Gonadotropin releasing hormone receptor p^*^ | 13 | 3.75 | 1.34x10^-4^ |
| Heterotrimeric G-protein signaling p^*^ | 113 | 3 | 0.41 | 8.21x10^-3^ | Wnt signaling pathway | 1 | 0.19 | 0.18 | Ionotropic glutamate receptor pathway | 6 | 0.81 | 1.87x10^-4^ |
| Metabotropic glutamate receptor g III | 56 | 2 | 0.2 | 1.78x10^-2^ | Gonadotropin releasing hormone receptor p^*^ | 0 | 0.15 | 0.86 | Alzheimer disease-presenilin pathway | 8 | 1.79 | 5.26x10^-4^ |
| 2-arachidonoylglycerol biosynthesis | 6 | 1 | 0.02 | 2.15x10^-2^ |  |  |  |  | Metabotropic glutamate receptor g III | 5 | 0.92 | 2.55x10^-3^ |
| Integrin signalling | 175 | 3 | 0.63 | 2.60x10^-2^ |  |  |  |  | Heterotrimeric G-protein signaling p^*^ | 7 | 1.86 | 2.98x10^-3^ |
| Interleukin signaling | 95 | 2 | 0.34 | 4.69x10^-2^ |  |  |  |  | Muscarinic acetylcholine receptor 1 and 3 signaling | 4 | 0.76 | 7.46x10^-3^ |
| Inflammation mediated by chemokine and cytokine signaling | 233 | 3 | 0.84 | 5.30x10^-2^ |  |  |  |  | Alpha adrenergic receptor signaling | 3 | 0.41 | 8.51x10^-3^ |
| Alzheimer disease-presenilin pathway | 109 | 2 | 0.39 | 5.98x10^-2^ |  |  |  |  | Histamine H1 receptor mediated signaling | 3 | 0.48 | 1.27x10^-2^ |

Abbreviations: Obs; Number of genes observed, Exp: Number of genes expected, p^*^: Pathway

**Supplementary Table 19.** Overall endometriosis and WHRadjBMI GRAIL results (P<0.5).

| **SNP** | **GRAIL P.Value** | **Candidate Genes** |
| --- | --- | --- |
| rs1884896 | 0.00155131 | *CDH4* |
| rs6860299 | 0.002823147 | *CDH9* |
| rs12681079 | 0.003374109 | *GATA4* |
| rs6865113 | 0.004806768 | *ISL1* |
| rs7914078 | 0.005194232 | *TCF7L2* |
| rs7251505 | 0.005421934 | *CEBPA* |
| rs12527010 | 0.007514152 | *HEY2* |
| rs3856226 | 0.010983828 | *BARHL2* |
| rs6556312 | 0.011331097 | *F12* |
| rs1800597 | 0.011738681 | *NTF3* |
| rs4695001 | 0.016999202 | *KCTD8* |
| rs7109584 | 0.023557656 | *GRIK4* |
| rs7935294 | 0.025248549 | *SPON1* |
| rs178642 | 0.031833826 | *MYH6* |
| rs9394816 | 0.038850722 | *FOXP4* |
| rs12672756 | 0.04064821 | *IGFBP1* |
| rs1035942 | 0.041069247 | *INSR* |
| rs7801581 | 0.050245498 | *EVX1* |
| rs1868894 | 0.053024171 | *F11* |
| rs6961094 | 0.053075949 | *CUX1* |
| rs3820282 | 0.055465195 | *WNT4* |
| rs701160 | 0.0579414 | *DISC1* |
| rs11101628 | 0.06825186 | *VENTX* |
| rs2236519 | 0.073315747 | *EYA2* |
| rs2921188 | 0.0807836 | *PPARG* |
| rs2860517 | 0.11192832 | *ETAA1* |
| rs9358819 | 0.12780715 | *C6orf32* |
| rs11975856 | 0.12985308 | *SCIN* |
| rs3734431 | 0.14624424 | *GCM1* |
| rs10096521 | 0.17220283 | *MYC* |
| rs6549200 | 0.19477808 | *FRMD4B* |
| rs11142949 | 0.19997249 | *TMEM2* |
| rs2782666 | 0.20897683 | *RUNX2* |
| rs2480211 | 0.23074961 | *CYB5R4* |
| rs1154245 | 0.23085773 | *LAMA3* |
| rs12214804 | 0.23826743 | *C6orf1* |
| rs9822326 | 0.28296197 | *LEKR1* |
| rs1250248 | 0.28653581 | *FN1* |
| rs12714287 | 0.28820387 | *ALK* |
| rs1898605 | 0.32003378 | *TRIB2* |
| rs13156373 | 0.32043679 | *ARL15* |
| rs7680787 | 0.33028121 | *ANXA5* |
| rs2918300 | 0.33111131 | *MGC33407* |
| rs10195252 | 0.33188499 | *COBLL1* |
| rs3936510 | 0.33244734 | *MAP3K1* |
| rs4017074 | 0.38266764 | *CDV3* |
| rs1795293 | 0.38969374 | *LSAMP* |
| rs9821836 | 0.39506405 | *ZNF385D* |
| rs2248556 | 0.4015406 | *LRRC7* |
| rs17431067 | 0.41220964 | *PLCB1* |

**Supplementary Table 20.** Stage B endometriosis and WHRadjBMI GRAIL results (P<0.5).

| **SNP** | **GRAIL P.Value** | **Candidate Genes** |
| --- | --- | --- |
| rs12097741 | 0.005063789 | *C1orf141* |
| rs13092529 | 0.008473136 | *ZNF385D* |
| rs2745359 | 0.008799377 | *RSPO3* |
| rs11060440 | 0.022837518 | *TMEM132D* |
| rs10428504 | 0.023836545 | *FAM13A1* |
| rs4791142 | 0.053326063 | *CCDC46* |
| rs1479605 | 0.064153465 | *TCF7L2* |
| rs12214804 | 0.064346222 | *C6orf1* |
| rs858031 | 0.074058519 | *KCNJ6* |
| rs3820282 | 0.074970382 | *WNT4* |
| rs2241490 | 0.085936672 | *BLID* |
| rs13156373 | 0.14059526 | *ARL15* |
| rs7602149 | 0.14166233 | *ACTR3* |
| rs4720174 | 0.14226363 | *TBX20* |
| rs7109584 | 0.14699399 | *GRIK4* |
| rs2782666 | 0.14748572 | *RUNX2* |
| rs10842708 | 0.15401581 | *ITPR2* |
| rs7637969 | 0.16234652 | *OSTN* |
| rs12527010 | 0.16443803 | *HEY2* |
| rs2408141 | 0.16860076 | *DBX2* |
| rs355900 | 0.17825353 | *COBLL1* |
| rs7680787 | 0.18365604 | *ANXA5* |
| rs9394816 | 0.19610761 | *FOXP4* |
| rs10878362 | 0.20016693 | *HMGA2* |
| rs1250248 | 0.21883538 | *FN1* |
| rs3861397 | 0.22533683 | *CITED2* |
| rs9307183 | 0.23357478 | *SHROOM3* |
| rs7406197 | 0.25150749 | *ABR* |
| rs2744372 | 0.25213981 | *DSP* |
| rs9406636 | 0.26098233 | *BNC2* |
| rs2285323 | 0.26236462 | *GAS7* |
| rs8192506 | 0.26391484 | *LOC130355* |
| rs11619804 | 0.27926838 | *MLNR* |
| rs17178718 | 0.2968041 | *NLRP1* |
| rs7801581 | 0.31877328 | *EVX1* |
| rs1535249 | 0.31985711 | *SLC24A3* |
| rs4657273 | 0.38215288 | *RGS5* |
| rs2436415 | 0.3832582 | *SH3PXD2B* |
| rs9999033 | 0.38942485 | *PDLIM3* |
| rs6961094 | 0.49513149 | *CUX1* |

**REFERENCES**

1 Painter, J.N., Anderson, C.A., Nyholt, D.R., Macgregor, S., Lin, J., Lee, S.H., Lambert, A., Zhao, Z.Z., Roseman, F., Guo, Q. *et al.* (2010) Genome-wide association study identifies a locus at 7p15.2 associated with endometriosis. *Nat. Genet.*, **43**, 51-54.

2 Speliotes, E.K., Willer, C.J., Berndt, S.I., Monda, K.L., Thorleifsson, G., Jackson, A.U., Allen, H.L., Lindgren, C.M., Luan, J., Magi, R. *et al.* (2010) Association analyses of 249,796 individuals reveal 18 new loci associated with body mass index. *Nat. Genet.*, **42**, 937-948.

3 Randall, J.C., Winkler, T.W., Kutalik, Z., Berndt, S.I., Jackson, A.U., Monda, K.L., Kilpelainen, T.O., Esko, T., Magi, R., Li, S. *et al.* (2013) Sex-stratified genome-wide association studies including 270,000 individuals show sexual dimorphism in genetic loci for anthropometric traits. *PLoS. Genet.*, **9**, e1003500.

4 Bajpai, A.K., Davuluri, S., Chandrashekar, D.S., Ilakya, S., Dinakaran, M. and Acharya, K.K. (2012) MGEx-Udb: a mammalian uterus database for expression-based cataloguing of genes across conditions, including endometriosis and cervical cancer. *PloS. One.*, **7**, e36776.

5 Grundberg, E., Small, K.S., Hedman, A.K., Nica, A.C., Buil, A., Keildson, S., Bell, J.T., Yang, T.P., Meduri, E., Barrett, A. *et al.* (2012) Mapping cis- and trans-regulatory effects across multiple tissues in twins. *Nat. Genet.*, **44**, 1084-1089.
